# Taxonomic Revision of Ningshan Odd-Scaled Snake, *Achalinus ningshanensis* (Serpentes, Xenodermidae), with Description of a New Subspecies from Western China †

**DOI:** 10.3390/ani14233425

**Published:** 2024-11-27

**Authors:** Yuhao Xu, Shun Ma, Bo Cai, Diancheng Yang, Tianyou Zhang, Tianxuan Gu, Fengcheng Zhu, Song Huang, Lifang Peng

**Affiliations:** 1 State Key Laboratory of Plateau Ecology and Agriculture, Qinghai University, Xining 810016, China; yuhao_xu@sinoophis.com (Y.X.);; 2 School of Ecological and Environmental Engineering, Qinghai University, Xining 810016, China; 3 Chengdu Institute of Biology, Chinese Academy of Sciences, Chengdu 610041, China; mashun21@mails.ucas.ac.cn (S.M.);; 4 University of Chinese Academy of Sciences, Beijing 100049, China; 5 College of Life and Environmental Sciences, Huangshan University, Huangshan 245021, China; 6 School of Plant Protection, Anhui Agricultural University, Hefei 230026, China; 7 School of Architecture and Environmental Art, Sichuan Fine Arts Institute, Chongqing 400053, China; 8 Anhui Province Key Laboratory of the Conservation and Exploitation of Biological Resource, College of Life Sciences, Anhui Normal University, Wuhu 241000, China

**Keywords:** *Achalinus ningshanensis occidentalis* ssp. nov., *Achalinus ningshanensis ningshanensis*, mitochondrial DNA, morphological characters, taxonomy, molecular phylogeny

## Abstract

*Achalinu ningshanensis* (Yang, Huang, Jiang, Burbrink, and Huang, 2022) was first described in Ningshan County, Shaanxi Province, China in 2022, based on seven female specimens. In this study, based on phylogenetic analyses using mitochondrial 12S ribosomal RNA (*12S*), 16S ribosomal RNA (*16S*), cytochrome c oxidase subunit 1 (*CO1*), cytochrome b (cyt *b*) gene fragments, and morphological examinations of specimens, we revise the taxonomic status of *A. ningshanensis*, and provide additional data on this species. The molecular phylogeny indicated that *A. ningshanensis* is nested in a highly supported monophyletic group, forming a sister taxon to *A. spinalis*, and is divided into two well-supported lineages, A and B, with an uncorrected *p*-distance between lineages from 3.6 to 4.3% for *CO1*. Therefore, we proposed that Lineage B from western Sichuan and southwestern Shaanxi is a new subspecies, *Achalinus ningshanensis occidentalis* ssp. nov., and Lineage A from southern Shaanxi and northeastern Sichuan is allocated as *Achalinus ningshanensis ningshanensis*. Morphologically, the new subspecies can be distinguished from its congeners, especially from *Achalinus ningshanensis ningshanensis*, by the following characteristics: (1) the tail is relatively short, with a TAL/TL ratio of 0.202–0.226 in males, and 0.155–0.178 in females; (2) there are two pairs of chin-shields; (3) there are 21–22 maxillary teeth; (4) the length of the suture between internasals is significantly shorter than that between prefrontals, with an LSBI/LSBP ratio of 0.502–0.773; (5) there are six supralabials, with the fourth and fifth in contact with the eye; (6) there are five to six infralabials, and the first to third or fourth touches the first pair of chin-shields; (7) there is one hexagonal loreal, with an LorH/LorL ratio of 0.612–1.040; (8) the two anterior temporals are in contact with the eye; (9) there are 155–160 ventrals in males, and 165–174 in females; (10) there are 60–65 subcaudals in males, and 49–53 in females, which are not paired; and (11) the dorsum is iridescent and uniformly charcoal black, lacks a longitudinal vertebral line, and has a dark brown or dark gray ventral area.

## Simple Summary

The odd-scaled snake genus *Achalinus* Peters, 1869 is widely distributed in northern Vietnam, China, and Japan, but is a group of snakes about which there is meager information. *Achalinu ningshanensis* was first described in 2022 and is only known from Ningshan County, Shaanxi Province, China. However, we detected a clear contradiction in the phylogenetic position between the type series and our newly collected topotypes. To solve this, we combined a mitochondrial phylogenetic analysis and morphological comparisons to revise the taxonomic status of *A. ningshanensis* in this study. Based on four mitochondrial combined gene fragments, molecular phylogenetic analyses indicated that *A. ningshanensis* is nested within a highly supported monophyletic group, forming a sister taxon to *A. spinalis*, which also revealed two well-supported lineages of *A. ningshanensis*. Based on morphology and phylogenetic methods, the lineage composed of the population from western Sichuan and southwestern Shaanxi represents a new subspecies, *Achalinus ningshanensis occidentalis* ssp. nov., and the other lineage represents the original species from southern Shaanxi and northeastern Sichuan, which we allocated as *Achalinus ningshanensis ningshanensis*. Finally, we provide a further discussion of the phylogenetic and taxonomic issues among the genus *Achalinus*.

## 1. Introduction

The genus *Achalinus* Peters, 1869, commonly named odd-scaled snakes due to their unique scutellation, represents a small-sized, cave-dwelling genus widely distributed across eastern and southeastern Asia, including northern Vietnam, China, and Japan [1,2,3,4,5]. Owing to their cryptic lifestyle, *Achalinus* species are difficult to detect in the wild. Consequently, for a considerable period, limited studies addressed the taxonomy, ecology, and natural history of *Achalinus*, and until 2019, this genus included only nine species [2,5]. With more comprehensive field sampling and DNA-barcoding deep efforts, the previously underestimated biodiversity of *Achalinus* has gradually been revealed, and in the past five years alone, at least 20 new species have been described [1,2,3,4,5,6,7,8,9,10,11,12,13,14,15]. Currently, this genus contains twenty-eight known species and two subspecies, with the majority found in China (twenty-one of twenty-eight) [15,16].

**Figure 1:**
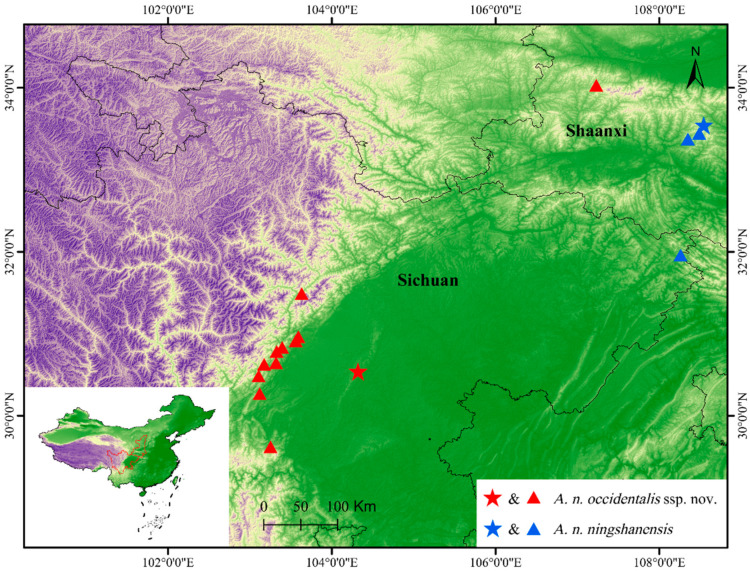
Known distribution of two subspecies of *Achalinus ningshanensis*: *Achalinus ningshanensis occidentalis* ssp. nov. (red star and red triangles) and *A. n. ningshanensis* (blue star and blue triangles). Stars represent the type of locality, and triangles represent the other known localities.

The Ningshan odd-scaled snake, *Achalinus ningshanensis* Yang, Huang, Jiang, Burbrink and Huang, 2022, was described based on seven female specimens from Ningshan County, Shaanxi Province, China [12]. It is mainly distinguished from its congeners by a combination of the following morphological characteristics: (1) the dorsum is uniformly dark brown and lacks a longitudinal vertebral line; (2) the venter surface is brown; (3) a dotted black streak in the middle of the subcaudals is lacking; (4) the tail length is relatively short, with a TAL/TL ratio of 0.12–0.16 in females; (5) there are fewer subcaudals, 41–46, in females; (6) 23 rows of dorsal scales throughout, which are strongly keeled, and the outer-most rows on both sides of the body are also keeled and slightly enlarged; (7) one loreal; (8) the internasal is not fused to prefrontal area; (9) the length of the suture between the internasals almost equal to that between the prefrontals; (10) preocular and postocular absent; (11) there are six supralabials; (12) there are five infralabials, and the first three (rarely, two) touch the first pair of chin-shields; (13) there are three pairs of chin-shields. During previous herpetological surveys, we collected six *Achalinus* specimens from Ningshan County, Shaanxi Province, and one specimen from Wanyuan City, Sichuan Province, China (Figure 1). Through morphological examinations, these specimens can undoubtedly be identified as *A. ningshanensis*. A phylogenetic analysis showed that the specimens from Ningshan County and Wanyuan City are nested within a highly supported monophyletic group, forming a sister taxon to *A. spinalis* (Peters, 1869). However, the molecular phylogenetic analysis provided by Yang et al. [12] showed that *A. ningshanensis* formed a sister group to *A. yangdatongi* Hou, Wang, Guo, Chen, Yuan and Che, 2021, which is clustered together with *A. juliani* Ziegler, Nguyen, Pham, Nguyen, Pham, Van Schingen, Nguyen and Le, 2019 and *A. ater* Bourret, 1937. The contradiction between the morphological and molecular results is undoubtedly confusing.

To address the phylogenetic divergence observed between the newly collected specimens and *A. ningshanensis*, we reached out to the original authors to re-sequence the type series of *A. ningshanensis*. However, due to the poor preservative conditions, we could not obtain high-quality PCR products from the tissues of all types of specimens to sequence and reestablish the phylogenetic relationship. Therefore, we conducted a detailed morphological examination of both the new specimens and the type series of *A. ningshanensis*. Our findings showed that the newly collected *Achalinus* specimens from Ningshan County and Wanyuan County matched the morphology of the type series, confirming their identification as *A. ningshanensis*. The discrepancies in the phylogenetic analysis appear to be sequencing errors in Yang et al. [12].

During the synchronous field work in 2024, we collected a total of 13 *Achalinus* specimens from western Sichuan Province, China. The phylogenetic analysis showed that these specimens were clustered within the same monophyletic group as the authentic sequences of *A. ningshanensis* but formed a distinct, well-supported lineage to *A. ningshanensis* within the clade. Morphologically, the *Achalinus* specimens from western Sichuan are distinguished from *A. ningshanensis*. Additionally, during our examination of specimens deposited in the collections of Anhui Normal University Museum (ANU), we found that a specimen collected from Taibai County, which was identified as *A. spinalis*, exhibited the same morphological characteristics as specimens from western Sichuan. Hence, in this study, based on the examination of museum material and newly collected *Achalinus* specimens, we reassessed the taxonomic status of *A. ningshanensis*, which was also supported by the data on molecular differentiation from the analyses of mitochondrial 12S ribosomal RNA (*12S*), 16S ribosomal RNA (*16S*), cytochrome c oxidase subunit 1 (*CO1*), and cytochrome *b* (cyt *b*) gene fragments. We provide additional data of this species and describe a new subspecies of *A. ningshanensis* from western Sichuan and southwestern Shaanxi Province, China.

## 2. Materials and Methods

### 2.1. Morphological Comparisons

A total of 32 specimens of the genus *Achalinus* were collected from 2021 to 2024 (Table 1). Sex was determined by tail dissection and by determining if hemipenes were present. After euthanasia with a lethal injection of a 0.7% tricaine methanesulfonate (MS-222, Changmao Biochemical Engineering Co., Ltd., Changzhou, China) solution, fresh liver tissue was extracted and immediately preserved in 95% ethanol. The specimens were fixed in 10% formaldehyde for one day, then transferred to 75% ethanol for permanent preservation, and deposited in Qinghai University Museum (**QHU**) and Chengdu Institute of Biology (**CIB**), Chinese Academy of Sciences. Sampling procedures involving live snakes were in accordance with the Wild Animals Protection Law of China and approved by the Institutional Ethics Committee of Qinghai University (protocol code SL-2023028). In addition, we also examined eight *Achalinus* specimens (including the type series of *A. ningshanensis*) deposited in Anhui Normal University Museum (**ANU**).

The terminology and methods of measurement characteristics and scalation counts followed Zhao [17], Ma et al. [13], and Xu et al. [18]. Bilateral morphological character measurements and scale feature counts were given as left/right. The utilization of abbreviations for morphological characteristics follows the conventions established by Darko et al. [19]. Three measurement characters were measured with a Deli Stainless Ruler (No. 8462) to the nearest 1 mm: **SVL** (snout–vent length), **TAL** (tail length), and **TL** (total length), and all other measurements were performed using Deli digital calipers (DL312200) to the nearest 0.1 mm: **HL** (head length) was taken from the tip of snout to the posterior margin of mandible; **HW** (head width) was measured around the widest part of the head in dorsal view; **LorH** (loreal height) was measured from the highest part to the lowest part of the loreal in the lateral view; **LorL** (loreal length) was measured from the most anterior loreal to the most posterior loreal in lateral view; **LSBI** (length of the suture between internasals); **LSBP** (length of the suture between prefrontals); **ED** (eye diameter) was taken from the anterior corner of the eye to the posterior corner. The scalation features are listed as follows: **SL** (supralabials); **IL** (infralabials); **IL-1st Chin** (infralabials touching the first pair of chin-shields); **Lor** (loreals); **PRO** (preoculars); **PO** (postoculars); **TEMP** (temporals); **aTEMP-Eye** (the number of anterior temporals touching the eye); **SPO** (supraoculars); **DSR** (dorsal scale rows), counted at one-head-length behind the head, at midbody, at one-head-length before the cloacal plate; **VS** (ventrals), counted from the anterior-most wide ventral scale after the chin-shields, following the midline, and concluding before the cloacal plate, **CP** (cloacal plate), and **SC** (subcaudals). The keeling states of the outer-most dorsal scale rows (**KOD**) were measured from the same positions as where we counted the dorsal scales. The number of the maxillary teeth (**MT**) was confirmed through scanning photography obtained by nano-computerized tomography (**CT**). Scans were performed using a GE V|tome|X m dual tube 300/180 kV system (developed by the Institute of High Energy Physics (IHEP), Chinese Academy of Sciences) at the Key Laboratory of Vertebrate Evolution and Human Origins, Institute of Vertebrate Paleontology and Paleoanthropology (IVPP), Chinese Academy of Sciences. The specimens were scanned with an energy beam of 80 kV and a flux of 80× μA using a 360° rotation and then reconstructed into the 4096 × 4096 matrices of 1536 slices. The final CT-reconstructed skull images were exported with a minimum resolution of 11.0 μm. Skull images were exported from the virtual 3D model reconstruction using Volume Graphics Studio ver. 3.4.0. Morphological comparison between species of the genus *Achalinus* were obtained from the specimens examined in this study and many key references [1,2,3,4,5,6,7,8,9,10,11,12,13,14,15,16,17,18,19,20,21,22,23,24,25,26,27,28,29,30,31,32,33].

Given the geographic and phylogenetic proximity between two populations of *A. ningshanensis*, we conducted statistical analyses on mensural and meristic characteristics using **R** v.4.3.2. The traits analyzed included HW/HL, LorH/LorL, LSBI/LSBP, TAL/TL, ED/HL, IL, Chin, VS, SC, and MT, which are the common diagnosis traits of *Achalinus*, with sexes compared separately due to sexual dimorphism in *Achalinus*. Morpho-spatial clustering and species/population positions were assessed using Principal Component Analysis (**PCA**) on a dataset of five meristic and five normalized morphometric characters, performed with the *prcomp* function and visualized using the *factoextra* package in R v.4.3.2.

### 2.2. Molecular Phylogeny

Genomic DNA was extracted from preserved liver tissues using QIAamp DNA Mini Kit (QIAGEN, Changsheng Biotechnology Co., Ltd., Changchun, China). Four mitochondrial gene fragments were targeted for phylogenetic analysis, including the 12S ribosomal RNA (*12S*), 16S ribosomal RNA (*16S*), cytochrome c oxidase subunit 1 gene (*CO1*), and cytochrome *b* (cyt *b*). PCR conditions followed previously reported protocols; *12S* was amplified by primers 12S2LM (5′-ACACACCGCCCGTCACCCT-3′)/16S5H (5′-CTACCTTTGCACGGTTAGGATACCGCGGC-3′) [34], *16S* was amplified by primers 16Sar_L (5′-CGCCTGTTTATCAAAAACAT-3′)/16Sbr-H (5′-CCGGTCTGAACTCAGATCACGT-3′) [35], *CO1* was amplified by primers Chmf4 (5′-TYTCWACWAAYCAYAAAGAYATCGG-3′)/Chmr4 (5′-ACYTCRGGRTGRCCRAARAATCA-3′) [36], and cyt *b* was amplified by primers L14910 (5′-GACCTGTGATMTGAAAACCAYCGTTGT-3′)/H16064 (5′-CTTTGGTTTACAAGAACAATGCTTTA-3′) [37]. PCR products were sequenced by Shanghai Map Biotech Co., Ltd. The raw sequences were assembled using SeqMan in the DNASTAR v.11.1 software package [38] and aligned in MEGA X [39]. The newly generated sequences have been submitted to GenBank (Table 1).

To explore the phylogenetic relationships, we used *12S*, *16S*, *CO1*, cyt *b* sequences from 14 species, along with *CO1* sequences from 12 additional species of the genus *Achalinus*. Homologous sequences from *Stoliczkia vanhnuailianai* Lalronunga, Lalhmangaiha, Zosangliana, Lalhmingliani, Gower, Das and Deepak, 2021 and *Xenodermus javanicus* Reinhardt, 1836 were used as outgroups. Since considerable species of the genus *Achalinus* currently only have *CO1* sequences available, we generated maximum likelihood (ML) phylogenetic trees based on both the *CO1* dataset and the concatenated *12S*/*16S*/*CO1*/cyt *b* dataset using IQ-TREE v1.6.12 [40], with node support evaluated through 5000 ultrafast bootstrap replicates (UFB), where UFB ≥ 95% was considered well-supported [41]. Additionally, single-branch support was assessed via the SH-like approximate likelihood ratio test (SH-aLRT) with 1000 replicates, considering nodes with SH ≥ 80% as well-supported [42]. Uncorrected pairwise distances (*p*-distance) among closely related congeners were calculated in MEGA X software [39]. All sequences were obtained from National Center for Biotechnology Information (NCBI) except the newly generated sequences. Bayesian posterior probabilities (BI, %) ≥ 95 were considered significantly supported.

## 3. Results

### 3.1. Phylogenetic Relationship

**Figure 2:**
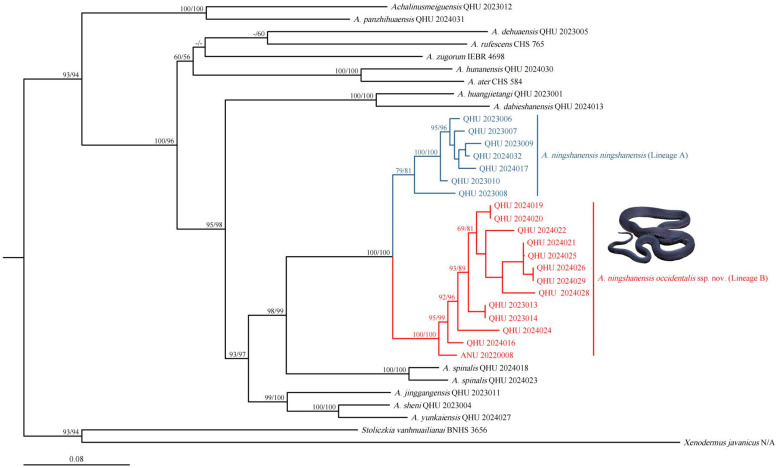
Maximum likelihood tree of the genus *Achalinus* inferred from four mitochondrial (*12S/16S*/*CO1*/cyt *b*) fragments. The nodes supporting values on branches are presented with the SH-like approximate likelihood ratio test (SH)/Ultrafast Bootstrap Approximation (UFB); the ones lower than 50 are displayed as “–”.

The concatenated sequence alignment was 2088 bp in length (*12S* = 318 bp; *16S* = 492 bp; *CO1* = 622 bp; cyt *b* = 656 bp). The topology obtained by the maximum likelihood analysis is shown in Figure 2: all putative species of *Achalinus* formed a highly supported lineage (SH 93/UFB 94). The specimens collected from Shaanxi Province and Sichuan Province formed a monophyletic group with well-supported values (SH 98/UFB 99) and were identified as the sister taxon to *A. spinalis*. The tree result was also consistent with the phylogenic relationship with two well-separated operational taxonomic units (OTUs): Lineage A and Lineage B. Lineage A contains specimens from southern Shaanxi and northeastern Sichuan, representing *A. ningshanensis ningshanensis*, while Lineage B comprises all samples from western Sichuan Province and southwestern Shaanxi Province, representing a potential subspecies of *A. ningshanensis*.

**Figure 3:**
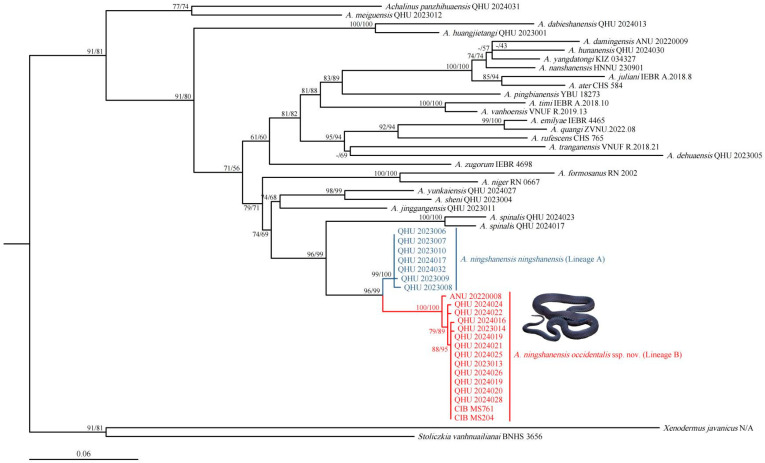
Maximum likelihood tree of the genus *Achalinus* inferred from *CO1* fragments. The nodes supporting values on branches are presented with the SH-like approximate likelihood ratio test (SH)/Ultrafast Bootstrap Approximation (UFB); the ones lower than 50 are displayed as “–”.

Based on the *CO1* gene (622 bp) alone, the phylogenetic tree constructed using maximum likelihood analysis showed that the samples of *Achalinus ningshanensis* formed a monophyletic group with two well-supported, deeply divergent clades, Lineage A and Lineage B (SH 96/UFB 99, Figure 3). The uncorrected *p*-distances between *A. ningshanensis* and other *Achalinus* species ranged from 8.8% (vs. *A. spinalis*) to 19.1% (vs. *A. panzhihuaensis* Hou, Wang, Guo, Chen, Yuan, and Che, 2021) (Table 2). The *p*-distance between Lineage A and Lineage B was 3.6% to 4.3%, which is close to the distances observed between certain *Achalinus* species, such as *A. nanshanensis* Li, Zhu, Xiao, Wu, Yang, Zhang, and Mo, 2024 vs. *A. yangdatongi* Hou, Wang, Guo, Chen, Yuan, and Che, 2021 (4.5%). The intraspecific genetic distances within Lineage A and Lineage B ranged from 0.0% to 0.5% and 0.0% to 0.7%, respectively.

**Table 1:** GenBank accession numbers, localities, and voucher information for all specimens used in this study.

Species Name	Locality	Voucher	*12S*	*16S*	*CO1*	*cyt b*	Reference
*A. n. occidentalis* ssp. nov.	Longquanyi, Sichuan, China	QHU 2023013	PQ509310	PQ505725	PQ507884	PQ515142	This study
*A. n. occidentalis* ssp. nov.	Longquanyi, Sichuan, China	QHU 2023014	PQ509311	PQ505726	PQ507885	PQ515143	This study
*A. n. occidentalis* ssp. nov.	Hongya, Sichuan, China	QHU 2024016	PQ509313	PQ505728	PQ507887	PQ515145	This study
*A. n. occidentalis* ssp. nov.	Aba, Sichuan, China	QHU 2024019	PQ509316	PQ505731	PQ507890	PQ515148	This study
*A. n. occidentalis* ssp. nov.	Chongzhou, Sichuan, China	QHU 2024020	PQ509317	PQ505732	PQ507891	PQ515149	This study
*A. n. occidentalis* ssp. nov.	Lushan, Sichuan, China	QHU 2024021	PQ509318	PQ505733	PQ507892	PQ515150	This study
*A. n. occidentalis* ssp. nov.	Lushan, Sichuan, China	QHU 2024022	PQ509319	PQ505734	PQ507893	PQ515151	This study
*A. n. occidentalis* ssp. nov.	Dayi, Sichuan, China	QHU 2024024	PQ509321	PQ505736	PQ507895	PQ515153	This study
*A. n. occidentalis* ssp. nov.	Qionglai, Sichuan, China	QHU 2024025	–	PQ505737	PQ507896	PQ515154	This study
*A. n. occidentalis* ssp. nov.	Qionglai, Sichuan, China	QHU 2024026	–	PQ505738	PQ507897	PQ515155	This study
*A. n. occidentalis* ssp. nov.	Chongzhou, Sichuan, China	QHU 2024028	PQ509323	PQ505740	PQ507898	PQ515157	This study
*A. n. occidentalis* ssp. nov.	Dayi, Sichuan, China	QHU 2024029	PQ509324	PQ505741	PQ507899	PQ515158	This study
*A. n. occidentalis* ssp. nov.	Mt. Qingcheng, Sichuan, China	CIB MS204	–	–	PQ507876	–	This study
*A. n. occidentalis* ssp. nov.	Dujiangyan, Sichuan, China	CIB MS761	–	–	PQ507877	–	This study
*A. n. occidentalis* ssp. nov.	Taibai, Shaanxi, China	ANU 20220008	NC_032084	NC_032084	MK064591	NC_032084	Li et al. [43]; Peng et al. [44]
*A. n. ningshanensis*	Ningshan, Shaanxi, China	QHU 2023006	PQ509303	PQ505718	PQ507879	PQ515135	This study
*A. n. ningshanensis*	Ningshan, Shaanxi, China	QHU 2023007	PQ509304	PQ505719	PQ507880	PQ515136	This study
*A. n. ningshanensis*	Wanyuan, Sichuan, China	QHU 2023008	PQ509305	PQ505720	PQ507881	PQ515137	This study
*A. n. ningshanensis*	Ningshan, Shaanxi, China	QHU 2023009	PQ509306	PQ505721	PP725557	PQ515138	Xu et al. [18]; This study
*A. n. ningshanensis*	Ningshan, Shaanxi, China	QHU 2023010	PQ509307	PQ505722	PP725558	PQ515139	Xu et al. [18]; This study
*A. n. ningshanensis*	Ningshan, Shaanxi, China	QHU 2024017	PQ509314	PQ505729	PQ507888	PQ515146	This study
*A. n. ningshanensis*	Ningshan, Shaanxi, China	QHU 2024032	PQ509328	PQ505745	PQ507902	PQ515162	This study
*A. ater*	China	CHS 584	–	MK194076	MK064760	MK201421	Li et al. [43]
*A. dabieshanensis*	Yuexi, Anhui, China	QHU 2024013	PQ509325	PQ505742	PQ507900	PQ515159	This study
*A. damingensis*	Nanning, Guangxi, China	ANU 20220009	–	–	OP644487	–	Yang et al. [5]
*A. dehuaensis*	Minhou, Fujian, China	QHU 2023005	PQ509302	PQ505717	PQ507878	PQ515134	This study
*A. emilyae*	Hoanh Bo, Vietnam	IEBR 4465	–	–	MK330857	–	Ziegler et al. [2]
*A. formosanus*	Taiwan, China	RN 2002	–	–	KU529452	–	Unpublished
*A. hunanensis*	Guizhou, China	QHU 2024030	PQ509326	PQ505743	PQ281493	PQ515160	This study
*A. huangjietangi*	Jinyun, Zhejiang, China	QHU2023001	PQ509312	PQ505727	PQ507886	PQ515144	This study
*A. jinggangensis*	Fujian, China	QHU 2023011	PQ509308	PQ505723	PQ507882	PQ515140	This study
*A. juliani*	Ha Lang, Cao Bang, Vietnam	IEBR A.2018.8	–	–	MK330854	–	Ziegler et al. [2]
*A. meiguensis*	Dayi, Sichuan, China	QHU 2023012	PQ509309	PQ505724	PQ507883	PQ515141	This study
*A. nanshanensis*	Huaihua, Hunan, China	HNNU 230901	–	–	OR523368	–	Li et al. [15]
*A. niger*	Taiwan, China	RN 0667	–	–	KU529433	–	Unpublished
*A. panzhihuaensis*	Panzhihua, Sichuan, China	QHU 2024031	PQ509327	PQ505744	PQ507901	PQ515161	This study
*A. pingbianensis*	Honghe, Yunnan, China	YBU 18273	–	–	MT365521	–	Li et al. [43]
*A. quangi*	Phu Yen, Son La, Vietnam	ZVNU.2022.08	–	–	OQ197471	–	Pham et al., 2023
*A. rufescens*	China	CHS 765	–	MK194205	MK064864	MK201515	Li et al. [43]
*A. sheni*	Shaoyang, Hunan, China	QHU 2023004			PP725556		Xu et al. [18]; This study
*A. spinalis*	Chongqing, China	QHU 2024018	PQ509315	PQ505730	PQ507889	PQ515147	This study
*A. spinalis*	Hubei, China	QHU 2024023	PQ509320	PQ505735	PQ507894	PQ515152	This study
*A. timi*	Thuan Chau, Son La, Vietnam	IEBR A.2018.10	–	–	MK330856	–	Ziegler et al. [2]
*A. tranganensis*	Ninh Binh, Vietnam	VNUF R.2018.21	–	–	MW023086	–	Luu et al. [7]
*A. vanhoensis*	Van Ho, Son La, Vietnam	VNUF R.2019.13	–	–	ON677935	–	Ha et al. [11]
*A. yangdatongi*	Wenshan, Yunnan, China	KIZ 034327	–	–	MW664865	–	Hou et al. [3]
*A. yunkaiensis*	Guizhou, China	QHU 2024027	PQ509322	PQ505739	PQ281492	PQ515156	This study
*A. zugorum*	Bac Me, Ha Giang, Vietnam	IEBR 4698	–	MT503100	MT502775	MT513238	Miller et al. [8]
**Out group**							
*Stoliczkia vanhnuailianai*	Mizoram, India	BNHS 3656	OL352693	OL352694	OL422476	OL422473	Deepak et al. [45]
*Xenodermus javanicus*	–	–	AF544781	AF544810	–	AF544810	Vidal and Hedges [46]
*Xenodermus javanicus*	Sumatera Barat, Indonesia	–	–	–	KP410747	–	Teynié et al. [47]

**Figure 4:**
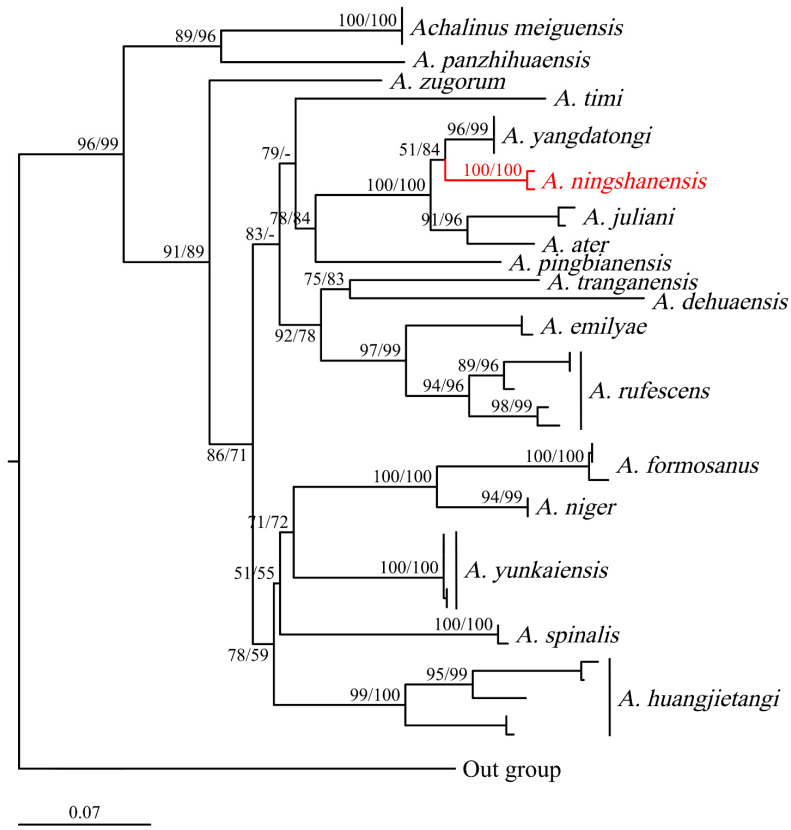
Reconstructed phylogenetic tree based on data from Yang et al. [12]. The nodes supporting values on branches are presented with the SH-like approximate likelihood ratio test (SH)/Ultrafast Bootstrap Approximation (UFB); the ones lower than 50 are displayed as “–”.

In Yang et al. [12], the molecular phylogenetic analysis showed that *A. ningshanensis* formed the sister group to *A. yangdatongi* Hou, Wang, Guo, Chen, Yuan and Che, 2021, and was then clustered together with *A. juliani* Ziegler, Nguyen, Pham, Nguyen, Pham, Van Schingen, Nguyen and Le, 2019 and *A. ater* Bourret, 1937 (Figure 4); however, this result appears inconsistent with actual phylogenetic relationships. We propose that the sequences ON548422 and ON548423 in Yang et al. [12] are erroneous. The accurate phylogenetic position and sequences of *A. ningshanensis* should be based on the findings in this study (Figure 2 and Figure 3; Table 1).

### 3.2. Morphological Results

**Figure 5:**
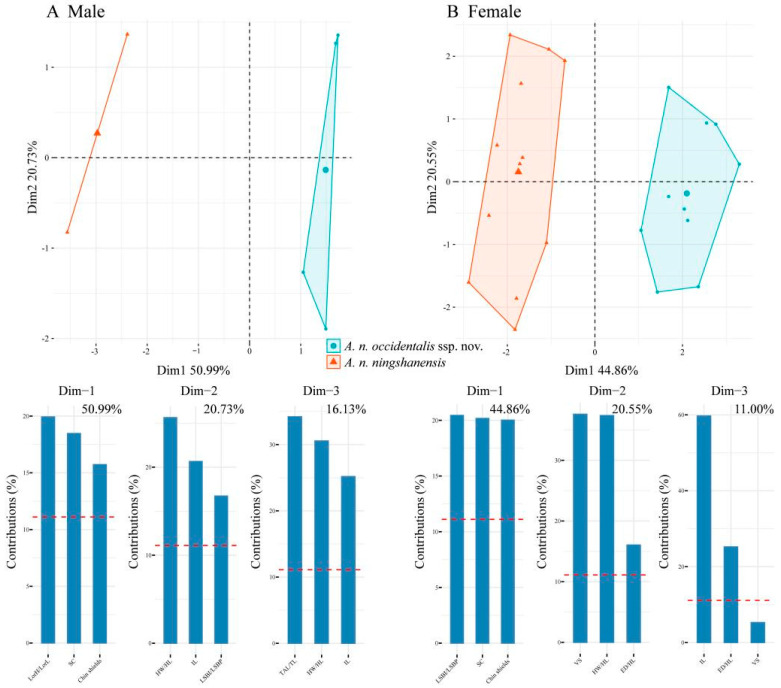
Male (**A**) and female (**B**) PCA plots between *Achalinus ningshanensis occidentalis* ssp. nov. and *A. n. ningshanensis* and bar plots of the percent contribution of each data type to Dim 1–3 of the PCA. The percentage score at the top of each bar plot is the percent contribution of that dimension to the overall variation in the dataset. The red dotted lines in the bar plots represent the mean percentage values.

The scatter plots of PC1 and PC2 for the two populations of *A. ningshanensis* indicate that, regardless of sex, the samples for each subspecies form distinct clusters with no overlap (Figure 5). In the PCA results, the first four principal components explained 95.92% of the total variation in males, with PC1, PC2, PC3, and PC4 accounting for 50.99%, 20.73%, 16.13%, and 8.07% of the variance, respectively. Similarly, for females, the first four components captured a substantial proportion of variation (85.07% in total), with PC1, PC2, PC3, and PC4 contributing 44.86%, 20.55%, 11.00%, and 8.66% of the variance, respectively. Considering these morphological distinctions and the unique phylogenetic position, we propose that the population from western China represents a new subspecies of *Achalinus ningshanensis*.

### 3.3. Taxonomy

*Achalinus ningshanensis ningshanensis* Yang, Huang, Jiang, Burbrink and Huang, 2022

Ningshan odd-scaled Snake/Níng Shǎn Jǐ Shé Zhǐ Míng Yà Zhǒng (宁陕脊蛇指名亚种)

**Figure 6:**
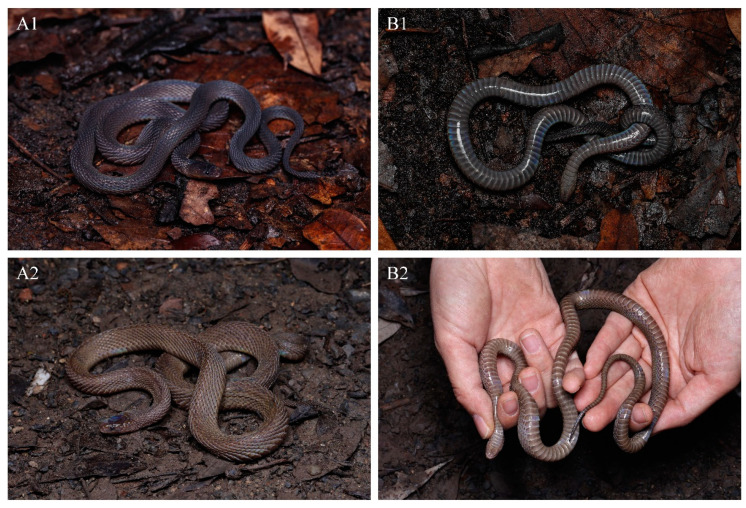
Dorsal (**A**) and ventral (**B**) views of living *Achalinus ningshanensis ningshanensis*. (**A1**,**B1**): QHU 2024017, male, from Ningshan County, Shaanxi Province; (**A2**,**B2**): QHU 2023009, female, from Ningshan County, Shaanxi Province. Photos by Yuhao Xu. Scale bars are not shown.

**Figure 7:**
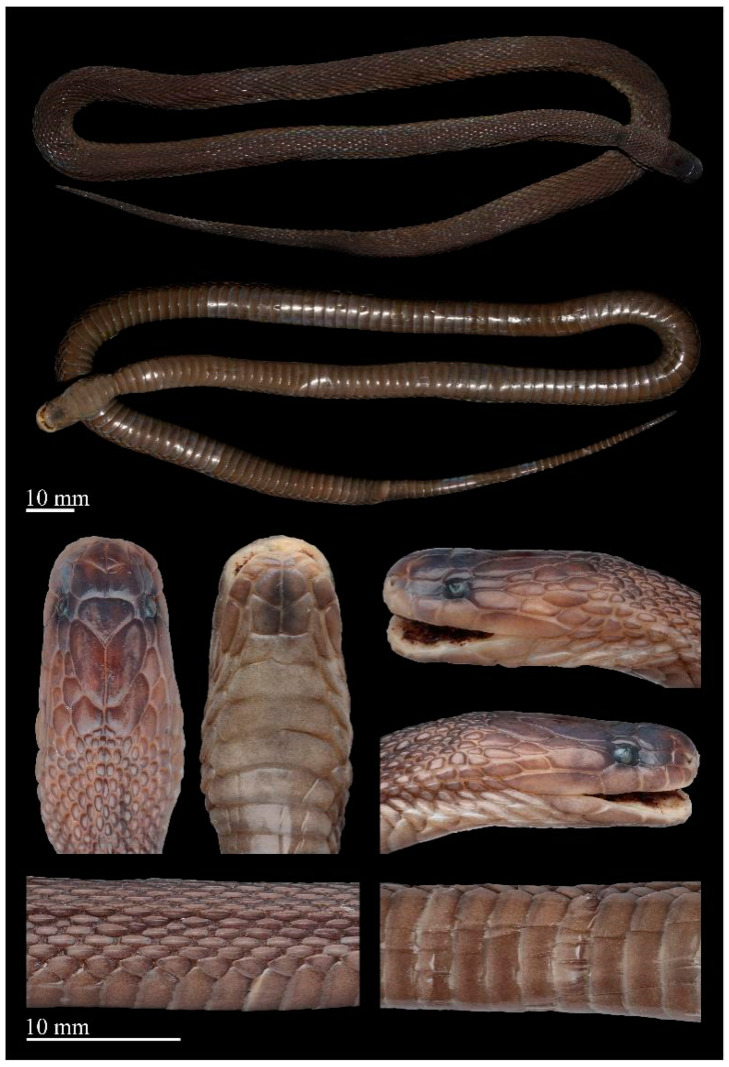
Preserved specimen of the holotype of *Achalinus ningshanensis ningshanensis* (ANU 20220001, female). Photos by Diancheng Yang and Yuhao Xu. Scale bars: 10 mm.

**Figure 8:**
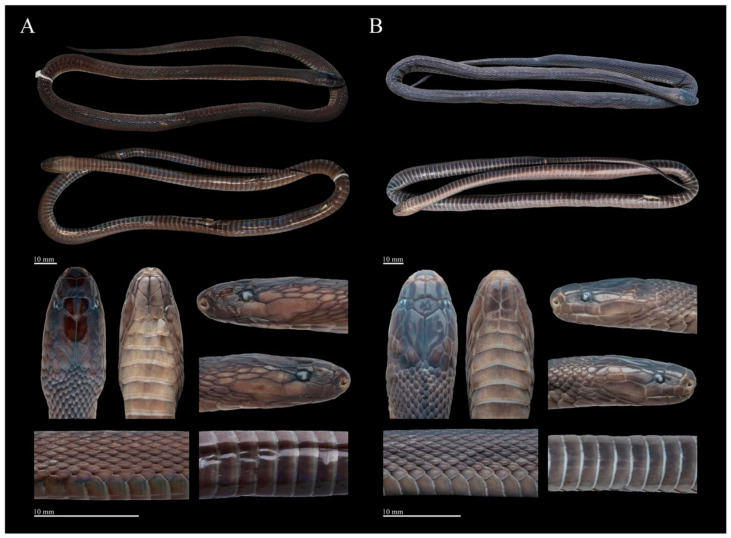
Preserved specimen of *Achalinus ningshanensis ningshanensis.* (**A**) QHU 2023008, adult male, from Wanyuan City, Sichuan Province; (**B**) QHU 2024032, adult female, topotype, from Ningshan County, Shaanxi Province. Photos by Yuhao Xu. Scale bars: 10 mm.

**Figure 9:**
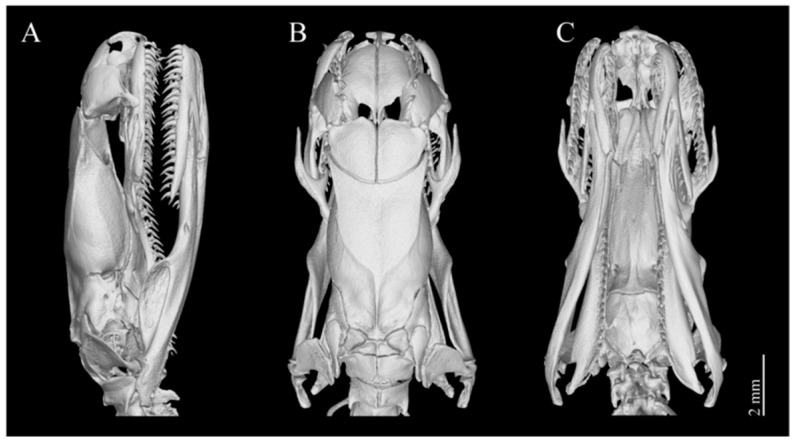
3D-reconstructed skull model of the holotype of *Achalinus ningshanensis ningshanensis* (ANU 20220001). (**A**) lateral view; (**B**) dorsal view; and (**C**) ventral view. Scale bars: 2 mm.

**Figure 15:**
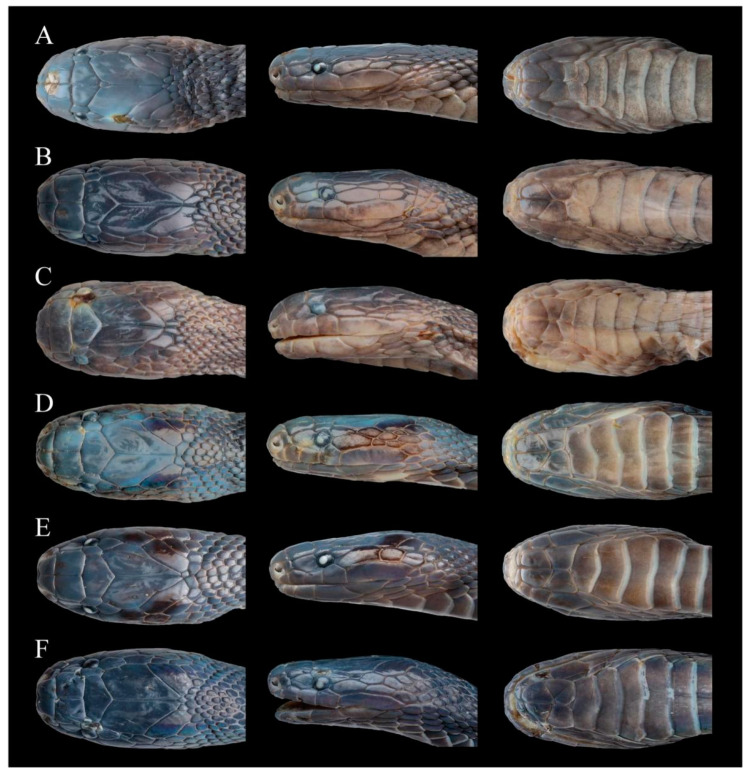
Dorsal (**left**), lateral (**middle**), and ventral (**right**) area of the head comparisons between *Achalinus ningshanensis ningshanensis* and *Achalinus ningshanensis occidentalis* ssp. nov. (**A**–**C**) *A. n. ningshanensis*: (**A**) QHU 2024017, adult male, from Ningshan County, Shaanxi Province; (**B**) QHU 2023006, adult female, from Ningshan County, Shaanxi Province; **C.** QHU 2023009, adult female, from Ningshan County, Shaanxi Province. (**D**–**F**) *A. n. occidentalis* ssp. nov.: (**D**) QHU 2023014, adult male, from Longquanyi District, Sichuan Province; (**E**) QHU 2023013, adult female, from Longquanyi District, Sichuan Province; and (**F**) QHU 2024022, adult female, from Lushan County, Sichuan Province. Photos by Yuhao Xu. Scale bars are not shown.

Figure 6, Figure 7, Figure 8, Figure 9 and Figure 15A–C

Chresonymy.

*Achalinus ningshanensis*: Li et al. [15]; Ma et al. [4]; Ma et al. [13]; Nguyen [48]; Xu et al. [18]; Yang et al. [5]; Yang et al. [12]; Zhang et al. [14].

*Achalinus* sp.: Xu et al. [18].

Examined specimens. A total of 14 specimens of *A. n. ningshanensis* were examined in this study. **Females** (n = 12): ANU 20220001–ANU 20220007, type series, collected by the team of Ke Jiang in summer of 2008 from Xunyangba Town, Ningshan County, Shaanxi Province, China (33°32′36.24″ N, 108°32′38.04″ E, 1372 m a. s. l.); QHU 2023006, QHU 2023007, QHU 2023009, and QHU 2023010, collected by the team of Lifang Peng in July of 2023 from Ningshan County, Shaanxi Province, China (33°26′40.2″ N, 108°29′04.56″ E, 1895 m a. s. l.); QHU 2024032, collected by Fengcheng Zhu, Zheming Zhang, and Fangyi Lai in September of 2024 from Chengguan Town, Ningshan County, Shaanxi Province, China (33°22′28.92″ N, 108°20′47.40″ E, 922 m a. s. l.). **Males** (n = 2): QHU 2023008, collected by Tianxuan Gu in July of 2023 from Mt. Yuquan, Wanyuan City, Sichuan Province, China (31°57′36.72″ N, 108°15′29.16″ E, 1453 m a. s. l.); QHU 2024017, collected by the team of Lifang Peng in April of 2024 from Ningshan County, Shaanxi Province, China (33°26′40.2″ N, 108°29′04.56″ E, 1895 m a. s. l.).

Revised diagnosis. *Achalinus ningshanensis ningshanensis* can be distinguished from its congeners by the following features: (1) the dorsum is uniformly brown to dark brown and lacks a longitudinal vertebral line; (2) the venter surface is brown; (3) the dotted black streak in the middle of the subcaudals is absent; (4) tail length is relatively short, with a TAL/TL ratio of 0.198–0.211 in males, and 0.121–0.161 in females; (5) fewer subcaudals, 51–56 in males, and 41–47 in females; (6) 23 rows of dorsal scales throughout, strongly keeled, and the outer-most rows on both sides of the body also keeled and slightly enlarged; (7) one loreal; (8) internasal not fused to prefrontal; (9) the length of the suture between internasals almost equal to that between prefrontals; (10) six supralabials; (11) four to five infralabials, with the first two or three touching the first pair of chin-shields; (12) three to four pairs of chin-shields; and (13) 20–22 maxillary teeth.

Description. Measurements and scalation data of *A. n. ningshanensis* specimens used in this study (n = 14) are presented in Table 3. The body is slender and cylindrical, TL 179–577 mm (363–412 mm in males, 179–577 mm in females); the tail is relatively short, with a TAL/TL ratio of 0.198–0.211 in males, and 0.121–0.161 in females; the head is slightly distinct from the neck, with an HW/HL ratio of 0.48–0.52 in males, and 0.37–0.57 in females; the eye is small, with an ED/HL ratio of 0.06–0.08 in males, and 0.07–0.10 in females; the rostrum is small, triangular, and slightly visible from above; the internasals are paired, and the length of the suture between the internasals is almost equal to that between the prefrontals, with an LSBI/LSBP ratio of 0.904–1.106; the nasal is divided into two sections by nasal cleft, with the nostrils in the anterior part of the nasal; the prefrontals are paired; the frontal area is pentagonal, pointed to the rear, slightly wider than high, and much shorter than the parietals; one loreal, subrectangular, LorH/LorL ratio 0.452–0.651; supraocular one, pentagonal; TEMP 7–9, arranged in three rows: (1–2) + (1–3) + (3–4), the anterior row contacts the eye; six supralabials, the fourth to fifth contact the eye, the last one is very elongated; three to four pairs of chin-shields; one mental; four to five infralabials, the first one in contact with each other after the mental area and before the first chin-shields. The first to third (rarely, second) touch the first pair of chin-shields.

Dorsal scale rows 23-23-23(21) are strongly keeled and lanceolate, and the outer-most row is also keeled and slightly enlarged. There are 161–165 in males vs. 159–177 in females; anal entire; SC 51–56 in males, and 41–47 in females, not paired.

Dentition. There are 20–22 maxillary teeth, without diastema, and they are nearly equal in length except for the first two, which are slightly smaller (Figure 9).

Coloration in life. In life, the dorsum is uniformly brown to dark brown with a metallic luster under light, and the interstitial skin of the dorsal area is dark brown. The ventral ground color is slightly lighter than that of the dorsum, and the posterior margins of ventral scales are light gray (Figure 6).

Coloration in preservation. In preservation, the coloration still resembles the specimen in life, except that the coloration of the dorsum deepens further, the background color of the venter becomes uniform light brownish, and posterior margins of ventral scales become grayish white (Figure 7 and Figure 8).

Distribution and natural history. *Achalinus ningshanensis ningshanensis* is currently known from Ningshan County, Ankang City, Shaanxi Province, and Wanyuan City, Dazhou City, Sichuan Province, China. Its known activity period spans from April to September, with peak activity occurring in the summer months. This species is typically found in grasslands, farmlands, and among stone piles near water sources at night, at elevations ranging from 800 to 1500 m. The surrounding habitat consists of secondary mixed forests of coniferous and broad-leaved trees (cf. this study, [12]).

Remarks. In 1983, Fang and Wang [49] reported a new provincial record for *Achalinus rufescens* in Shaanxi Province, based on two female specimens (voucher number 60,013 and 80019) collected from Hu County (now Huyi District, Xi’an City, at an altitude of 1400 m) and Ningshan County (at an altitude of 1500 m), Shaanxi Province. The main morphological characteristics were as follows: TL 517 + 77 mm in specimen 60013, and 400 + 70 mm in specimen 80019; the rostrum is triangular, its width is greater than its height, and is slightly visible from above; the internasals are paired, the length of the suture between the internasals is significantly shorter than that between the prefrontals, with the LSBI/LSBP ratio of 0.5; SL 6, the first one is the smallest, and the last one is very elongated; IL 5, the first one contact with each other after the mental; chin-shields 3/2; DSR 23-23-23, lanceolate and strongly keeled, the outer-most row smooth; VS 171–175; SC 43–46, not paired; CP complete. Based on the described morphology and collection locations, we suspect these specimens may actually be misidentified as *A. ningshanensis*. However, certain characteristics, such as the suture between internasals being much shorter than that between the prefrontals, the smooth outer-most dorsal scale row, and chin-shields 3/2, differ from *A. ningshanensis ningshanensis*. Since we have not been able to directly examine these specimens, we retained this record as it currently stands.

*Achalinus ningshanensis occidentalis* ssp. nov. Xu, Ma, Yang, Cai, Huang and Peng, 2024

http://zoobank.org/urn:lsid:zoobank.org:act:75A7E98C-EAB7-4C42-B02D-D0BAE362E3D9 (accessed on 2 November 2024)

**Figure 10:**
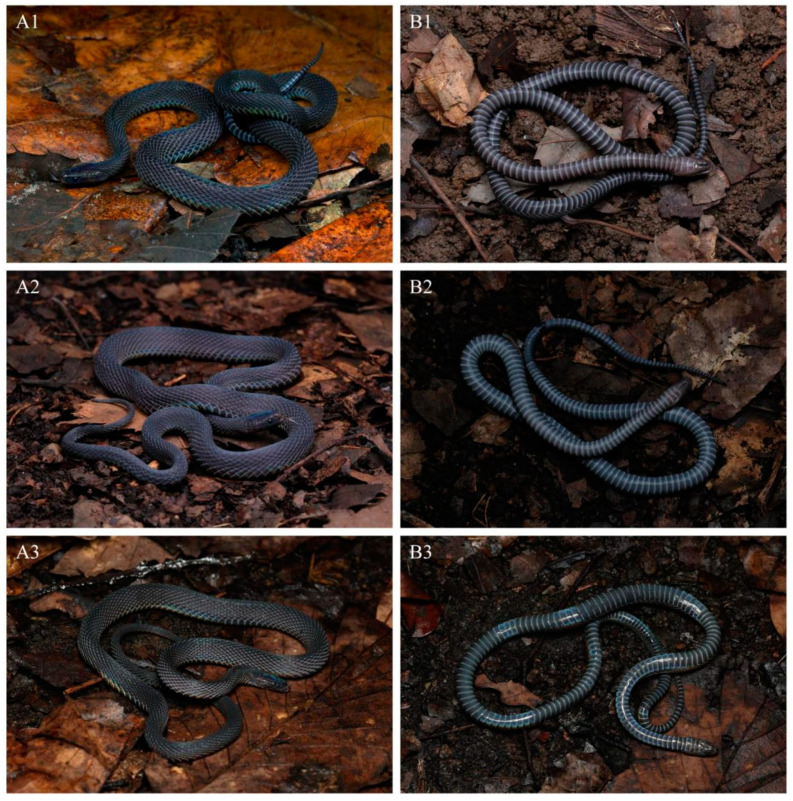
Dorsal (**A**) and ventral (**B**) views of *Achalinus ningshanensis occidentalis* ssp. nov. in life. (**A1**,**B1**): QHU 2023013, holotype, adult female, from Longquanyi District, Sichuan Province; (**A2**,**B2**): QHU 2023014, paratype, adult male, from Longquanyi District, Sichuan Province; (**A3**,**B3**): QHU 2024016, paratype, adult male, from Hongya County, Sichuan Province. Photos by Yuhao Xu.

**Figure 11:**
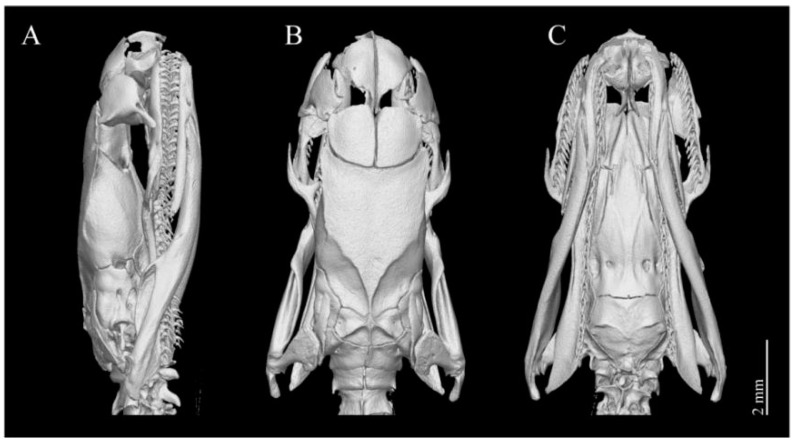
3D-reconstructed skull model of the paratype of *Achalinus ningshanensis occidentalis* ssp. nov. (QHU 2023014). (**A**) lateral view; (**B**) dorsal view; and (**C**) ventral view. Scale bars: 2 mm.

**Figure 12:**
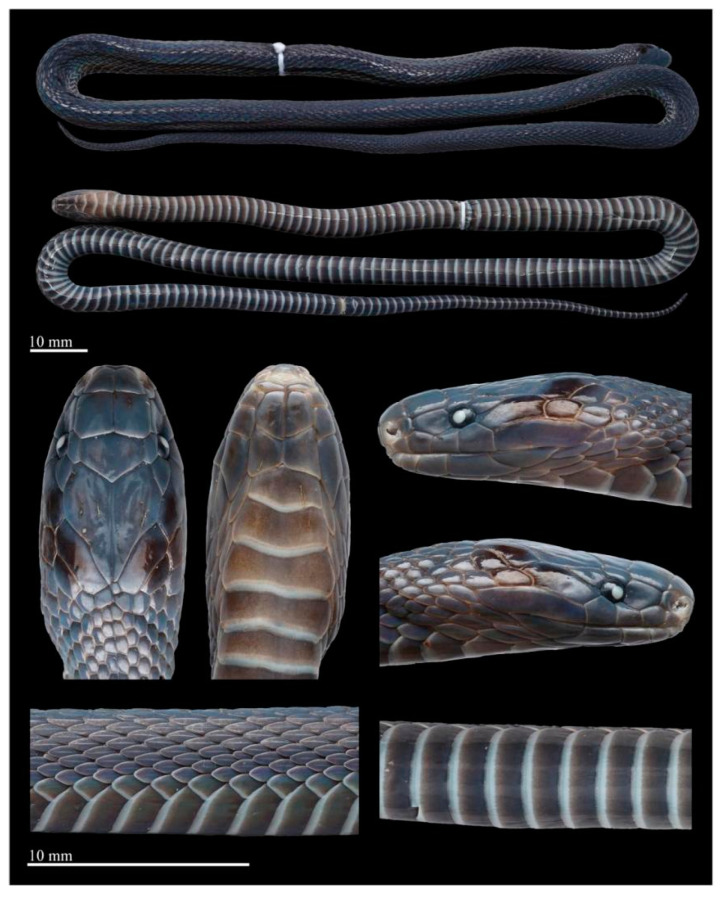
Preserved specimen of the holotype of *Achalinus ningshanensis occidentalis* ssp. nov. (QHU 2023013, adult female). Photos by Yuhao Xu. Scale bars: 10 mm.

**Figure 13:**
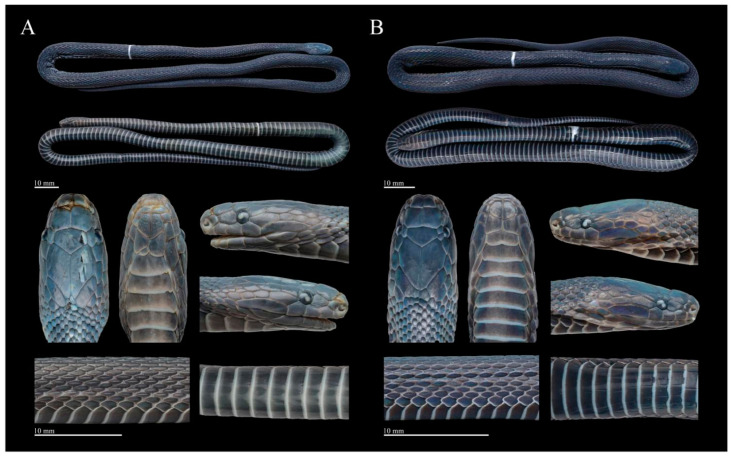
Preserved specimen of the paratypes of *Achalinus ningshanensis occidentalis* ssp. nov. (**A**) QHU 2024016, adult male, from Hongya County, Sichuan Province; (**B**) QHU 2024093, subadult female, from Dayi County, Sichuan Province. Photos by Yuhao Xu. Scale bars: 10 mm.

**Figure 14:**
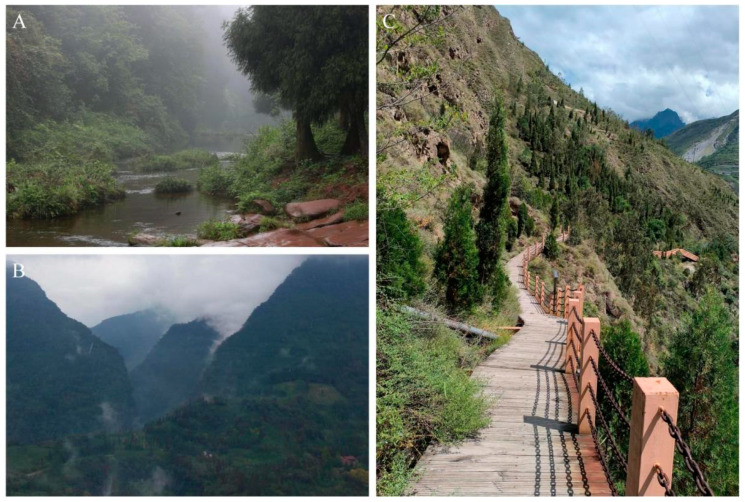
Habitats of *Achalinus ningshanensis occidentalis* ssp. nov. (**A**) Mt. Tiantai, Qionglai City, Sichuan Province, photo by Tianxuan Gu; (**B**) Lushan County, Yaan City, Sichuan Province, photo by Bo Cai; and (**C**) Wenchuan County, Aba Tibetan and Qiang Autonomous Prefecture, Sichuan Province, photo by Maozhou Xu.

**Figure 16:**
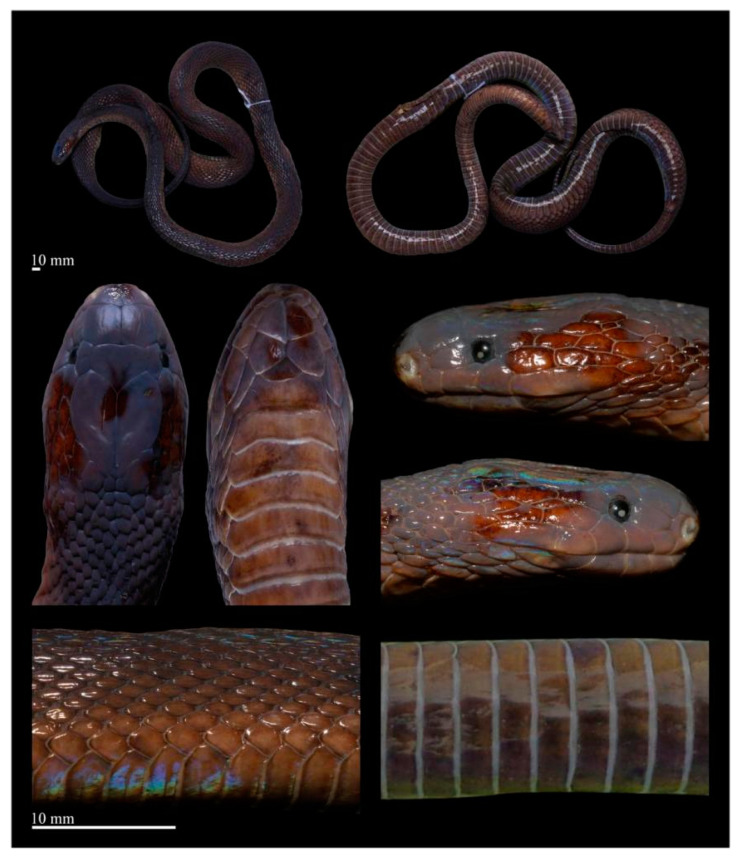
Preserved specimen of the ANU 20220008 (adult female, paratype of *Achalinus ningshanensis occidentalis* ssp. nov., from Taibai County, Shaanxi Province). Photos by Diancheng Yang. Scale bars: 10 mm.

Figure 10, Figure 11, Figure 12, Figure 13, Figure 14, Figure 15D–F and Figure 16

Chresonymy.

*Achalinus spinalis*: Zhao et al. [50]; Peng et al. [44]; Ziegler et al. [2]; Li et al. [6]; Luu et al. [7]; Miller et al. [8]; Li et al. [10]; Li et al. [43].

*Achalinus* sp.: Yang et al. [5]; Xu et al. [18].

Holotype. QHU 2023013, an adult female, collected by the team of Lifang Peng on 25 September, 2023 from Longquanyi district, Chengdu City, Sichuan Province, China (30°32′19.67″ N, 104°19′18.12″ E; 845 m a.s.l.)

Paratypes. **Females** (n = 9): QHU 2024020, adult, collected on 6 May 2024 from Chongzhou City, Chengdu City, Sichuan Province, China (30°47′08.16″ N, 103°19′32.88″ E; 1024 m a.s.l.); QHU 2024021, QHU 2024022, adult, collected in May 2024 from Lushan County, Yaan City, Sichuan Province, China (30°29′10.68″ N, 103°6′15.48″ E; 1107 m a.s.l.); QHU 2024025, QHU 2024026, adult, collected by Maozhou Xu and Tianxuan Gu on 31 May 2024 from Mt. Tiantai, Qionglai City, Sichuan Province, China (30°16′12.36″ N, 103°07′07.68″ E; 1300 m a.s.l.); QHU 2024028, adult, collected on 18 June 2024 from Chongzhou City, Chengdu City, Sichuan Province, China (30°50′00.06″ N, 103°23′30.73″ E; 1232 m a.s.l.); and QHU 2024029, subadult, collected on 18 June 2024 from Dayi County, Chengdu City, Sichuan Province, China (30°38′45.96″ N, 103°19′01.92″ E; 1030 m a.s.l.) by the team of Lifang Peng. CIB MS761, adult, collected by Bo Cai on 5 August 2024 from Dujiangyan City, Chengdu City, Sichuan Province, China (30°57′43.92″ N, 103°35′24.00″ E; 750 m a.s.l.). ANU 20220008 (also CHS 007 in Li et al. [43], and HUM201200001 and HS 12093 in Peng et al. [44]), adult, collected by the team of Song Huang on 05 August, 2012 from Taibai County, Baoji City, Shaanxi Province, China (34°01′51.59″ N, 107°13′41.16″ E; 1677 m a.s.l.). **Males** (n = 4): QHU 2023014, adult, with the same collected information as the holotype; QHU 2024016, adult, ollected on 21 April 2024 from Hongya County, Meishan City, Sichuan Province, China (29°37′17.75″ N, 103°15′2.88″ E; 737 m a.s.l.); QHU 2024019, subadult, collected on 29 April 2024 from Wenchuan County, Aba Tibetan and Qiang Autonomous Prefecture, Sichuan Province, China (31°29′20.40″ N, 103°37′53.76″ E; 1400 m a.s.l.); and QHU 2024024, juvenile, collected on 11 June 2024 from Dayi County, Chengdu City, Sichuan Province, China (30°37′42.96″ N, 03°10′19.20″ E; 1300 m a.s.l.) by the team of Lifang Peng.

Diagnosis. (1) the tail is relatively short, with a TAL/TL ratio of 0.202–0.226 in males, and 0.155–0.178 in females; (2) there are two pairs of chin-shields; (3) there are 21–22 maxillary teeth; (4) the length of the suture between internasals is significantly shorter than that between prefrontals, with the LSBI/LSBP ratio of 0.502–0.773; (5) there are six supralabials, and the fourth and fifth are in contact with the eye; (6) there are five to six infralabials, and the first to third or fourth touch the first pair of chin-shields; (7) there is one loreal, hexagonal, with an LorH/LorL ratio of 0.612–1.040; (8) the two anterior temporals are in contact with eye; (9) there are 155–160 ventrals in males, and 165–174 in females; (10) there are 60–65 subcaudals in males, and 49–53 in females, not paired; (11) the dorsum is iridescent and uniformly charcoal black, lacks a longitudinal vertebral line, and the ventral area is dark brown or dark gray.

Description of the holotype. Measurements and scalation. The adult female had a TL of 367 mm (SVL 302 mm and TAL 65 mm), complete tail, and TAL/TL ratio of 0.177; the body was slender and cylindrical; the head was slightly distinct from the neck, with an HW of 5.95 mm, HL of 13.12 mm, and HW/HL ratio of 0. 47; the eye is small; ED of 0.8 mm; the rostrum is small, triangular, and slightly visible from above; the length of the suture between the internasals is significantly shorter than that between the prefrontals, LSBI of 1.12 mm, LSBP of 1.71 mm, and LSBI/LSBP ratio of 0.654; one supraocular, hexagonal, width significantly greater than height, in contact with loreal, prefrontal, frontal, parietal, and the upper anterior temporal; prefrontals paired; frontal single, pentagonal, pointing to the rear, the width slightly larger than the length; one loreal, hexagonal, LorH 1.07 mm, LorL 1.22 mm, LorH/LorL ratio 0.877; TEMP 6, arranged in three rows (2 + 1 + 3); the anterior pair is elongated, in contact with the eye, the upper anterior temporal is small, the lower anterior temporal is large, in contact with fifth and sixth supralabials; SL 6/6, the first one is the smallest, the fourth and fifth are in contact with the eye, and the sixth is the longest; two pairs of chin-shields; one mental area; IL 5/5, the first three touching the first pair of chin-shields.

Dorsal scale rows 21-22-21, strongly keeled and lanceolate, the outer-most row slightly enlarged, smooth in the anterior part and the middle of the body and keeled toward the posterior section. VS 167; SC 50, not paired; CP entire.

Dentition. A total of 21 maxillary teeth, without diastema, nearly equal in length except for the first two, which are slightly smaller (Figure 13).

Coloration of holotype in life. The dorsum, including the head, body, and tail, is charcoal black with a slight iridescent sheen and lacks a longitudinal vertebral line. The dorsal head scales match the dorsum in color, while the interstitial skin and scale sutures are light gray. The mental and chin-shields are dark brown, and the gular region is a lighter brown. The anterior part of the ventral surface of the body is dark brown, gradually deepening toward the posterior, and the ventral surface of the tail is almost black. The posterior margins of the ventral scales are grayish-white (Figure 10(A1,B1)).

Coloration of holotype in preservation. In preservation, the coloration still resembles the specimen in life, except that the coloration of the ventral area is lightened (Figure 12).

Variation. The main morphological characters of *Achalinus ningshanensis occidentalis* ssp. nov. are listed in Table 4. The paratypes exhibit a similar morphological pattern to the holotype, but the majority of paratypes have dorsal scale rows in 23-23-23. Moreover, there is pronounced sexual dimorphism: compared to females, the examined males have a significantly long tail, TAL/TL ratio of 0.202–0.226 (vs. 0.155–0.178 in females), fewer ventrals (155–160 vs. 165–174 in females), and more subcaudals (60–65 vs. 49–53 in males).

Etymology. The subspecific epithet “*occidentalis*”, meaning “western”, signifies that the new subspecies is found in western China (western Sichuan and southwestern Shaanxi), with a range lying west of *A. n. ningshanensis*. We suggest “Western China Odd-scaled Snake” as its English common name and “Níng Shǎn Jǐ Shé Huá Xī Yà Zhŏng (宁陕脊蛇华西亚种)” as its Chinese common name.

Distribution and natural history. *Achalinus ningshanensis occidentalis* ssp. nov. is currently known to be distributed in western China, including Sichuan Province (Longquanyi District, Chongzhou City, Dayi County, Qionglai City, Dujiangyan City, Mt. Qingcheng, Hongya County, Lushan County, and Wenchuan County) and Shaanxi Province (Taibai County). Its known activity period spans from April to September, with peak activity occurring in early summer. Its activity period spans from April to September, with peak activity in early summer. This species is nocturnal and lives in subtropical mountainous regions at elevations of 737–1677 m, typically found within the leaf litter of subtropical broadleaf forests, mixed conifer–broadleaf forests, or coniferous forests, as well as in grasslands, farmland, or rock piles (Figure 14). Notably, specimen QHU 2024019 was discovered in a dry-hot valley in Wenchuan County, indicating a possible adaptation to diverse microhabitats within its range.

Comparisons. *Achalinus ningshanensis occidentalis* ssp. nov. can be separated from *A. n. ningshanensis* as follows: (1) LSBI is significantly shorter than LSBP (vs. suture between internasals is similar size when compared to the suture between prefrontals); (2) two pairs of chin-shields (vs. three or four pairs); (3) VS 155–160 in males (vs. 161–165 in males); (4) SC 60–65 in males, 49–53 in females (vs. 51–56 in males, 41–47 in females; (5) loreal is relatively high, LorH/LorL ratio of 0.760–0.946 in males, and 0.612–1.040 in females (vs. LorH/LorL ratio of 0.544–0.617 in males, 0.458–0.681 in females), and (6) dorsum is uniformly charcoal black (vs. uniformly brown to dark brown dorsum) (Figure 15, Table 5). A detailed comparison between *Achalinus ningshanensis occidentalis* ssp. nov. and its congeners is summarized in Table 6.

*Achalinus ningshanensis occidentalis* ssp. nov. can be separated from *A. huangjietangi*, *A. dabieshanensis* and *A. spinalis* due to the lack of a longitudinal vertebral line and fewer infralabials (usually 5 vs. 6). Moreover, it can be separated from *A. spinalis* due to having more maxillary teeth (21–22 vs. 16–20) and from *A. dabieshanensis* due to having more subcaudals in males (60–65 vs. 46–55) and more ventrals (155–174 vs. 141–155).

By the internasal separated from prefrontal, *Achalinus ningshanensis occidentalis* ssp. nov. can be separated from *A. meiguensis* Hu and Zhao, 1966 and *A. panzhihuaensis* Hou, Wang, Guo, Chen, Yuan & Che, 2021 (vs. internasal fused to prefrontal). Furthermore, it can be separated from *A. meiguensis* due to having 23 (rarely 22) DSR at the midbody (vs. 19–21), and from *A. panzhihuaensis* due to having fewer subcaudals in males (60–65 vs. 73), and 23 (rarely 21) DSR on the posterior body (vs. 19).

By LSBI/LSBP < 1, *Achalinus ningshanensis occidentalis* ssp. nov. can be easily separated from *A. ater* Bourret, 1937, *A. damingensis* Xu, Yang, Wu, Gong, Huang and Huang, 2023, *A. dehuaensis* Li, Wu, Xu, Zhu, Ren, Guo and Dong, 2021, *A. emilyae* Ziegler, Nguyen, Pham, Nguyen, Pham, van Schingen, Nguyen and Le, 2019, *A. hunanensis* Ma, Shi, Xiang, Shu and Jiang, 2023, *A. jinggangensis* (Zong and Ma, 1983), *A. juliani* Ziegler, Nguyen, Pham, Nguyen, Pham, van Schingen, Nguyen and Le, 2019, *A. nanshanensis*, *A. quangi* Pham, Pham, Le, Ngo, Ong, Ziegler and Nguyen, 2023, *A. rufescens*, *A. timi* Ziegler, Nguyen, Pham, Nguyen, Pham, Van Schingen, Nguyen and Le, 2019, *A. tranganensis* Luu, Ziegler, Ha, Lo, Hoang, Ngo, Le, Tran and Nguyen, 2020, *A. yangdatongi* Hou, Wang, Guo, Chen, Yuan and Che, 2021, *A. vanhoensis* Ha, Ziegler, Sy, Le, Nguyen and Luu, 2022, and *A. zugorum* Miller, Davis, Luong, Do, Pham, Ziegler, Lee, De Queiroz, Reynolds and Nguyen, 2020 (vs. >1), and from *A. hainanus* Huang, 1975, *A. sheni* Ma, Xu, Qi, Wang, Tang, Huang and Jiang, 2023, *A. werneri* Van Denburgh, 1912, and *A. yunkaiensis* Wang, Li and Wang, 2019 (vs. =1). Moreover, it can be distinguished from *A. damingensis*, *A. dehuaensis*, *A. hunanensis*, *A. juliani*, *A. nanshanensis*, *A. quangi*, *A. timi*, *A. tranganensis*, *A. yangdatongi*, *A. vanhoensis*, and *A. zugorum* due to it having fewer subcaudals (49–65 vs. 74 in *A. damingensis*, 74–81 in *A. dehuaensis*, 69–72 in *A. hunanensis*, 81–91 in *A. juliani*, 64–77 in *A. nanshanensis*, 69–84 in *A. quangi*, 72 in *A. timi*, 73+ in *A. tranganensis*, 76–82 in *A. yangdatongi*, 84 in *A. vanhoensis*, and 70 in *A. zugorum*). It can be separated from *A. hainanus* due to it having fewer subcaudals in females (49–53 vs. 67–69), and a comparatively shorter tail length in females (TAL/TL ratio 0.155–0.178 vs. 0.258–0.266). It can be separated from *A. sheni* due to it having fewer ventrals in males (155–160 vs. 161–170). It can be separated from *A. werneri* due to it having a comparatively shorter tail (TAL/TL ratio 0.155–0.226 vs. 0.250–0.300). It can be separated from *A. yunkaiensis* due to it having more subcaudals (60–65 vs. 49–56) and a comparatively longer tail length (0.202–0.226 vs. 0.185–0.200) in males, and more ventrals (166–174 vs. 144–156) in females.

By the loreal separated from prefrontal, *Achalinus ningshanensis occidentalis* ssp. nov. can be separated from *A. formosanus chigirai* Ota & Toyama, 1989, *A. f. formosanus* Boulenger, 1908, and *A. pingbianensis* Li, Yu, Wu, Liao, Tang, Liu & Guo, 2020 (vs. loreal fused to prefrontal).

Remarks. In 2017, Peng et al. [44] provided the mitochondrial genome of an *Achalinus* specimen (voucher number CHS 007 in Li et al. [43], HUM 201200001 and HS 12093 in Peng et al. [44], and ANU 20220008 in this study) from Taibai County, Shaanxi Province, China, which was identified as *A. spinalis*. The specimen was initially deposited at Huangshan University and was then transferred to Anhui Normal University Museum (ANU). This specimen has since been included in many studies of the genus *Achalinus*, where its original identification has been consistently used [1,2,3,6,7,8,43]. In 2023, Yang et al. [5], based on a phylogenetic analysis, reclassified it as *Achalinus* sp., but without providing any morphological description. In the present study, we examined this specimen (Figure 16, Table 4) and found the morphology of this specimen to be consistent with that of *Achalinus ningshanensis occidentalis* ssp. nov. Subsequent phylogenetic analyses further support the morphology results. Thus, we re-identify the specimen ANU 20220008 here as *Achalinus ningshanensis occidentalis* ssp. nov.

## 4. Discussion

*Achalinus spinalis* was once considered to be widely distributed across eastern and southeastern Asia, from Laos, Vietnam (Lao Cai, Vinh Phuc, and Thai Nguyen Province) and China (Jiangsu, Zhejiang, Anhui, Fujian, Jiangxi, Hubei, Hunan, Guangxi, Sichuan, Guizhou, Yunnan, Shaanxi, and Gansu Province) to Japan (Kyushu Island, Honshu Island, Koshiki Island, and Tokuno-shima Island) [17,51,52,53,54,55,56,57,58,59,60]. However, recent studies have shown that in various regions, the currently recognized *A. spinalis* has not formed a monophyletic group, with significant genetic divergence between lineages [1,2,3,4,5,6,7,8,9,10,11,12,13,14,15]. This suggests the *A. spinalis* sensu stricto may be a range-restricted species that is only found in Japan, and the taxonomic status of the populations recognized in other regions need to be re-evaluated. Through taxonomic work, several populations previously identified as *A. spinalis* have been described as new species (e.g., *A. huangjietangi*, *A. dabieshanensis* and *A. sheni*), while some erroneous distribution records have been gradually corrected [4,9,13,48]. However, due to the lack of genetic information of *A. spinalis* from the type locality, there is a gap remaining about the classification and evolutionary history within this species.

*Achalinus spinalis weigoldi* Mell, 1909 was described by Mell [23] based on the specimen form “Wa Shan, eastern Szechwan”; then, it was synonymized with *Achalinus spinalis* by Pope [52]. The original description of *Achalinus spinalis weigoldi* is as follows: “V 160–167, a 162; Sc 63–67, a 65; 3 pair of chin-shields; Sq (mid-dorsal scale rows) 21; length 600 mm”. Since Mell [23] did not designate the type of specimens or mention where the specimens were deposited, we can only compare the morphology of *Achalinus ningshanensis occidentalis* ssp. nov. with the few provided characteristics of *Achalinus spinalis weigoldi* in the original description. Morphologically, *Achalinus ningshanensis occidentalis* ssp. nov. can be separated from *Achalinus spinalis weigoldi* due to it having two pairs of chin-shields (vs. three), twenty-three mid-dorsal scale rows, rarely twenty-two (vs. twenty-one), and a significantly smaller body size (178–493+ mm vs. 600 mm). Additionally, the real type locality of *Achalinus spinalis weigoldi* cannot be confirmed either. In 2024, Nguyen et al. [48] discussed the taxonomic issues within the genus *Achalinus*. For *Achalinus spinalis weigoldi*, the authors suggested that the type locality “Wa Shan”, likely corresponds to Mt. Wawu, as mentioned by Inger et al. [61]. They also identified the specimen of *A. spinalis* from Inger et al. [61] as *A.* cf. *weigoldi*. However, “Wa Shan” in Sichuan has historically referred to at least two different mountains, Mt. Wawu (in Hongya County, Meishan City) and Mt. Dawa (in Leshan City), which are at least 50 km apart. Since Mell [23] did not provide a detailed description of the location, it is impossible to accurately determine the type locality of *Achalinus spinalis weigoldi*. Consequently, we suppose that *Achalinus spinalis weigoldi* Mell, 1909 is a *nomen nudum* and exclude it from formal use.

Clear species boundaries are essential for effective biodiversity conservation planning and action [62,63,64,65,66,67,68]. In this study, we re-evaluated the systematic position of *A. ningshanensis* and described the populations, which were previously identified as *A. spinalis*, from southwestern Shaanxi and western Sichuan Province, as *Achalinus ningshanensis occidentalis* ssp. nov. As for morphology, *Achalinus ningshanensis occidentalis* ssp. nov. can be identified from *A. n. ningshanensis* according to the fact that the LSBI is significantly shorter than the LSBP (vs. suture between internasals is similar size when compared to the suture between prefrontals); there are two pairs of chin-shields (vs. three or four pairs); VS 155–160 in males (vs. 161–165 in males); SC 60–65 in males, 49–53 in females (vs. 51–56 in males, 41–47 in females; and the dorsum is uniformly charcoal black (vs. a uniformly brown to dark brown dorsum). In terms of molecular systematics, the population from western Sichuan and southwestern Shaanxi forms two distinct, moderately divergent lineages with the population from southern Shaanxi and northeastern Sichuan Province, with an uncorrected *p*-distance from 3.6% to 4.3% in *CO1*. Although the distances are comparable to some other recognized species, i.e., *A. nanshanensis* vs. *A. yangdatongi* (*CO1* 4.4%) [15], *A. quangi* vs. *A. emilyae* (*CO1* 4.0–4.7%) [33], we currently conservatively consider this distinct population as a subspecies of *A. ningshanensis*. Isolation factors, such as geographic barriers, are likely critical in restricting the gene flow between populations, potentially driving genetic divergence and speciation events. Future research requires broader surveys combined with ecological and genetic data to understand these isolation mechanisms and explore the role of isolation and connectivity in evolution and conservation [69,70,71].

## 5. Conclusions

We revised the taxonomic status of *Achalinus ningshanensis*, and described a new subspecies, *Achalinus ningshanensis occidentalis* ssp. nov., based on ten female and four male specimens collected from western Sichuan and south western Shaanxi Province, China. Due to their cryptic lifestyle, the discovery of the new subspecies is largely accidental, which makes it difficult for us to make accurate judgments on the distribution and population status of this species. Further research is needed to elucidate the true distribution range and ecological niche of the new subspecies. Broader surveys in this area are also essential to gather additional genetic information from various populations, along with phylogenomic analyses to understand evolutionary patterns and assess the potential gene flow between these populations, ultimately clarifying species boundaries.

## Figures and Tables

**Table 2:** Uncorrected *p*-distance (%) among the *Achalinus* species based on partial mitochondria *CO1* gene.

ID	Species	1–15	16–22	23	24	25	26	27	28	29	30	31	32	33
**1–15**	*A. n. occidentalis* ssp. nov.	0.0–0.7												
**16–22**	*A. n. ningshanensis*	3.6–4.3	0.0–0.5											
**23**	*A. ater*	16.3–16.4	15.1–15.8	–										
**24**	*A. dabieshanensis*	15.8–16.3	15.8–16.5	17.6	–									
**25**	*A. damingensis*	16.5–16.9	14.3–14.9	8.4	18.7	–								
**26**	*A. dehuaensis*	17.4–18.0	16.7–16.9	19.4	21.0	18.5	–							
**27**	*A. emilyae*	14.7–15.3	14.6–15.1	13.4	20.6	15.0	16.8	–						
**28**	*A. meiguensis*	16.8–17.1	15.8–16.0	17.5	20.4	19.4	20.1	17.0	–					
**29**	*A. formosanus*	15.8–16.0	14.3–14.7	16.4	22.0	17.2	17.9	15.8	17.2	–				
**30**	*A. huangjietangi*	15.6–15.8	14.7–15.0	15.3	9.4	17.2	17.6	16.7	16.5	19.3	–			
**31**	*A. hunanensis*	17.1–17.5	15.1–15.5	7.5	20.1	7.1	18.5	14.5	18.4	16.7	17.8	–		
**32**	*A. jinggangensis*	11.4–11.6	9.4–9.8	14.4	16.7	13.7	16.0	14.6	14.1	13.3	14.5	13.3	–	
**33**	*A. juliani*	16.5–17.0	14.2–14.8	7.2	19.0	9.1	17.2	14.4	18.6	14.0	16.8	9.5	14.0	–
**34**	*A. nanshanensis*	16.0–16.0	14.9–15.6	7.0	19.5	5.9	16.5	15.2	20.3	16.5	18.2	5.8	14.3	8.4
**35**	*A. niger*	12.5–13.1	11.2–11.6	15.1	17.6	16.2	19.4	13.7	14.8	9.7	16.7	15.7	12.9	13.9
**36**	*A. panzhihuaensis*	18.9–19.1	17.6–18.0	18.4	18.3	19.8	20.8	17.7	12.5	20.2	16.3	19.1	17.6	18.6
**37**	*A. pingbianensis*	14.4–15.0	11.7–11.9	12.7	17.3	11.9	17.8	14.4	18.7	16.5	16.2	12.5	12.8	13.2
**38**	*A. quangi*	14.5–15.1	14.4–14.8	13.6	21.1	15.0	16.9	3.3	17.1	16.3	16.7	13.9	15.0	14.4
**39**	*A. rufescens*	16.9–17.4	15.5–15.7	14.0	19.0	14.6	14.7	10.3	20.9	16.4	16.5	13.3	14.2	12.5
**40**	*A. sheni*	11.8–12.3	10.5–10.7	14.8	17.8	15.9	16.4	15.5	14.7	14.6	16.7	14.6	10.4	15.9
**41–42**	*A. spinalis*	10.6–11.3	8.8–9.6	16.7–16.9	17.2–18.0	16.0–16.9	16.3–16.5	15.5–16.6	17.7–17.9	15.4	14.9–16.9	15.9–16.0	11.6–11.8	15.6–15.8
**43**	*A. timi*	14.8–15.1	13.6–14.1	15.0	18.9	15.1	19.1	15.1	18.1	15.1	17.8	14.6	15.3	16.1
**44**	*A. tranganensis*	15.2–15.7	14.7–15.3	14.6	17.6	16.2	16.5	13.3	17.5	19.7	15.0	15.6	16.2	15.6
**45**	*A. vanhoensis*	15.8–16.0	14.1–14.5	14.8	18.8	14.1	17.6	13.7	18.7	15.9	17.3	13.2	13.4	15.4
**46**	*A. yangdatongi*	15.0–15.2	13.2–13.9	6.5	19.6	5.9	15.6	14.4	19.2	16.6	15.8	5.7	13.0	7.7
**47**	*A. yunkaiensis*	11.4–12.4	11.0–11.4	14.1	18.0	14.5	16.1	15.1	16.9	13.3	15.6	13.9	10.2	13.9
**48**	*A. zugorum*	15.2–15.6	13.1–13.5	15.7	16.9	14.4	16.3	14.1	16.6	14.9	14.9	13.7	13.0	15.0
**ID**	**Species**	**34**	**35**	**36**	**37**	**38**	**39**	**40**	**41–42**	**43**	**44**	**45**	**46**	**47**
**34**	*A. nanshanensis*	–												
**35**	*A. niger*	14.7	–											
**36**	*A. panzhihuaensis*	19.0	16.6	–										
**37**	*A. pingbianensis*	12.9	13.2	19.2	–									
**38**	*A. quangi*	15.0	13.1	19.3	15.6	–								
**39**	*A. rufescens*	13.1	15.9	18.2	14.1	11.1	–							
**40**	*A. sheni*	16.0	14.1	17.1	12.9	16.6	14.9	–						
**41–42**	*A. spinalis*	15.9–16.4	15.2–15.6	18.9–19.6	13.8	15.0–15.7	14.2–14.8	11.8–13.0	3.2					
**43**	*A. timi*	15.7	13.1	18.4	12.9	15.1	16.6	15.5	15.8–16.6	–				
**44**	*A. tranganensis*	15.2	16.4	18.4	14.6	13.6	13.8	15.3	16.0–16.6	15.9	–			
**45**	*A. vanhoensis*	14.3	14.3	17.5	11.8	13.9	15.6	15.6	13.9–14.8	5.4	14.9	–		
**46**	*A. yangdatongi*	4.5	15.5	17.5	12.5	14.2	12.9	15.5	15.1–15.3	14.8	14.4	12.5	–	
**47**	*A. yunkaiensis*	13.3	13.7	17.1	13.1	15.3	13.6	6.9	12.4	15.5	13.9	13.8	12.9	–
**48**	*A. zugorum*	14.8	14.8	17.2	11.7	14.8	16.1	11.9	13.6–14.6	15.2	13.4	13.2	13.8	12.7

**Table 3:** Main morphological characteristics of *Achalinus ningshanensis ningshanensis* obtained from specimens examined in this study. K + K + K = keeled on anterior, mid, and posterior body.

Voucher Number	QHU 2023006	QHU 2023007	QHU 2023008	QHU 2023009	QHU 2023010	QHU 2024017	QHU 2024032
**Location**	Ningshan, Shaanxi	Ningshan, Shaanxi	Wanyuan, Sichuan	Ningshan, Shaanxi	Ningshan, Shaanxi	Ningshan, Shaanxi	Ningshan, Shaanxi
**Sex**	Female	Juvenil female	Male	Female	Female	Male	Female
**TL**	577	179	363	571	428	412	472
**TAL**	83	28	72	89	67	87	70
**TAL/TL**	0.144	0.156	0.198	0.156	0.157	0.211	0.148
**HW**	7.8	4.7	6.1	8.3	6.9	6.5	7.2
**HL**	16.3	8.7	12.7	14.5	13.7	12.4	13
**ED**	1.33	0.84	0.7	1.29	1.01	1.0	1.3
**MT**	20	21	20	21	20	20	22
**SL**	6/6	6/6	6/6	6/6	6/6	6/6	6/6
**IL**	4/4	4/5	5/5	5/5	5/5	5/5	5/5
**Chin**	3/3	3/3	3/3	3/3	3/3	4/3	4/4
**IL-1st Chin**	1st-3rd/1st-3rd	1st-3rd/1st-3rd	1st-3rd/1st-3rd	1st-3rd/1st-3rd	1st-3rd/1st-3rd	1st-3rd/1st-3rd	1st-3rd/1st-3rd
**Lor**	1	1	1	1	1	1	1
**LorH**	0.98	0.68	0.80	1.27	1.11	0.95	0.91
**LorL**	2.07	1.12	1.47	1.95	1.63	1.54	1.53
**LorH/LorL**	0.473	0.607	0.544	0.651	0.681	0.617	0.595
**LSBI/LSBP**	=	=	=	=	=	=	=
**TEMP**	2 + 2 + 4/2 + 2 + 3	2 + 2 + 3/2 + 2 + 3	2 + 3 + 3/2 + 3 + 3	2 + 3 + 4/2 + 2 + 4	1 + 2 + 4/1 + 2 + 3	2 + 2 + 4/2 + 2 + 4	2 + 1 + 3/2 + 1 + 3
**ATEMP-Eye**	2	2	2	2	2	2	2
**DSR**	23-23-23	23-23-23	23-23-23	23-23-23	23-23-23	23-23-23	23-23-23
**KOD**	K + K + K	K + K + K	K + K + K	K + K + K	K + K + K	K + K + K	K + K + K
**VS**	175	175	165	176	168	161	177
**SC**	47	47	51	47	45	56	44
**CP**	1	1	1	1	1	1	1
**Voucher Number**	**ANU 20220001**	**ANU 20220002**	**ANU 20220003**	**ANU 20220004**	**ANU 20220005**	**ANU 20220006**	**ANU 20220007**
**Location**	Ningshan, Shaanxi	Ningshan, Shaanxi	Ningshan, Shaanxi	Ningshan, Shaanxi	Ningshan, Shaanxi	Ningshan, Shaanxi	Ningshan, Shaanxi
**Sex**	Female	Female	Female	Female	Female	Female	Female
**TL**	436	491	527	416	398	450	430
**TAL**	62	72	64	63	64	69	65
**TAL/TL**	0.142	0.147	0.121	0.151	0.161	0.153	0.151
**HW**	7.16	7.48	4.75	6.27	5.5	5.94	4.79
**HL**	13.72	13.32	12.69	11.74	12.35	11.17	11.47
**ED**	1.12	1.19	/	1.1	1.12	0.82	0.96
**MT**	22	21	21	22	/	/	/
**SL**	6/6	6/6	6/6	6/6	6/6	6/6	6/6
**IL**	5/5	5/5	5/5	5/5	5/5	5/5	5/5
**Chin**	3/3	3/3	3/3	3/3	3/3	3/3	3/3
**IL-1st Chin**	1st-3rd/1st-3rd	1st-3rd/1st-3rd	1st-3rd/1st-3rd	1st-2nd/1st-2nd	1st-3rd/1st-3rd	1st-3rd/1st-3rd	/
**Lor**	1	1	1	1	1	1	1
**LorH**	0.97	0.91	0.93	1.04	0.88	0.83	/
**LorL**	1.90	2.01	1.71	1.8	1.92	1.59	/
**LorH/LorL**	0.511	0.452	0.544	0.578	0.458	0.522	/
**LSBI/LSBP**	=	=	=	=	=	=	=
**TEMP**	2 + 3 + 4/2 + 3 + 4	2 + 3 + 4/2 + 3 + 4	2 + 2 + 4/2 + 2 + 4	2 + 2 + 3/2 + 2 + 3	2 + 2 + 4/2 + 2 + 4	2 + 2 + 3/2 + 2 + 3	2 + 2 + 4/2 + 2 + 4
**ATEMP-Eye**	2	2	2	/	/	/	/
**DSR**	23-23-23	23-23-23	23-23-23	23-23-23	23-23-23	23-23-23	23-23-21
**KOD**	K + K + K	K + K + K	K + K + K	K + K + K	K + K + K	K + K + K	K + K + K
**VS**	167	174	171	170	159	174	164
**SC**	43	46	43	43	45	45	41
**CP**	1	1	1	1	1	1	1

**Table 4:** Main morphological characteristics of *Achalinus ningshanensis occidentalis* ssp. nov. obtained from specimens examined in this study. S + S + K = smooth on anterior and mid, keeled on posterior body; S + K + K = smooth on anterior, keeled on mid and posterior body.

Voucher Number	QHU 2023013	QHU 2023014	QHU 2024016	QHU 2024019	QHU 2024020	QHU 2024021	QHU 2024022
	Holotype	Paratype	Paratype	Paratype	Paratype	Paratype	Paratype
**Location**	Longquanyi, Sichuan	Longquanyi, Sichuan	Hongya, Sichuan	Wenchuan, Sichuan	Chongzhou, Sichuan	Lushan, Sichuan	Lushan, Sichuan
**Sex**	Female	Male	Male	Subadultmale	Female	Female	Female
**TL**	367	364	421	280	408	440+	403
**TAL**	65	81	95	60	71	64+	64
**TAL/TL**	0.177	0.223	0.226	0.214	0.174	/	0.159
**HW**	5.6	5.8	6.3	5.0	6.0	5.9	6.1
**HL**	12.0	12.4	12.5	9.3	12.4	13.4	10.8
**ED**	0.8	0.9	1.56	0.87	1.0	1.2	1.2
**MT**	21	21	21	21	21	22	21
**SL**	6/6	6/6	6/6	6/6	6/6	6/6	6/6
**IL**	5/5	5/5	5/6	5/5	5/5	5/5	5/5
**Chin**	2/2	2/2	2/2	2/2	2/2	2/2	2/2
**IL-1st Chin**	1st-3rd/1st-3rd	1st-3rd/1st-3rd	1st-3rd/1st-4th	1st-3rd/1st-3rd	1st-3rd/1st-3rd	1st-3rd/1st-3rd	1st-3rd/1st-3rd
**Lor**	1	1	1	1	1	1	1
**LorH**	1.07	1.17	1.41	1.22	1.33	1.62	1.23
**LorL**	1.22	1.54	1.60	1.29	1.57	1.85	1.46
**LorH/LorL**	0.877	0.760	0.881	0.946	0.847	0.876	0.842
**LSBI/LSBP**	0.654	0.599	0.722	0.773	0.640	0.657	0.771
**TEMP**	2 + 1 + 3/2 + 1 + 3	2 + 2 + 3/2 + 2 + 3	2 + 2 + 4/2 + 2 + 3	2 + 2 + 3/2 + 2 + 4	2 + 2 + 3/2 + 2 + 3	2 + 2 + 3/2 + 2 + 3	2 + 2 + 3/2 + 2 + 4
**ATEMP-Eye**	2/2	2/2	2/2	2/2	2/2	2/2	2/2
**DSR**	21-22-21	23-23-23	23-23-23	23-23-23	23-23-23	23-23-23	23-23-23
**KOD**	S + S + K	S + K + K	S + K + K	S + S + K	S + S + K	S + S + K	S + S + K
**VS**	167	155	160	158	171	165	171
**SC**	50	60	61	65	50	43+	49
**CP**	1	1	1	1	1	1	1
**Voucher** **Number**	**QHU 2024024**	**QHU 2024025**	**QHU 2024026**	**QHU 2024028**	**QHU 2024029**	**CIB MS761**	**ANU 20220008**
	**Paratype**	**Paratype**	**Paratype**	**Paratype**	**Paratype**	**Paratype**	**Paratype**
**Location**	Dayi, Sichuan	Qionglai, Sichuan	Qionglai, Sichuan	Chongzhou, Sichuan	Dayi, Sichuan	Dujiangyan, Sichuan	Taibai, Shaanxi
**Sex**	Juvenil male	Female	Female	Female	Subadult female	Female	Female
**TL**	178	409	455	366	309	387	493+
**TAL**	36	71	79	65	48	69	86+
**TAL/TL**	0.202	0.174	0.174	0.178	0.155	0.178	/
**HW**	4.1	5.4	6.4	5.6	5.4	6.2	8.55
**HL**	8.2	12.3	12.4	11.4	10.3	11.2	16.94
**ED**	0.70	1.11	1.18	1.07	0.9	/	1.47
**MT**	/	22	21	21	22	21	/
**SL**	6/6	6/6	6/6	6/6	6/6	6/6	6/6
**IL**	6/5	5/5	6/6	5/5	6/5	5/5	5/5
**Chin**	2/2	2/2	2/2	2/2	2/2	2/2	2/2
**IL-1st Chin**	1st-4th/1st-3rd	1st-3rd/1st-3rd	1st-3rd/1st-3rd	1st-3rd/1st-3rd	1st-3rd/1st-3rd	1st-3rd/1st-3rd	1st-3rd/1st-3rd
**Lor**	1	1	1	1	1	1	1
**LorH**	0.97	1.07	1.29	1.51	1.05	1.12	1.62
**LorL**	1.09	1.43	1.63	1.45	1.64	1.83	2.38
**LorH/LorL**	0.890	0.748	0.791	1.040	0.640	0.612	0.681
**LSBI/LSBP**	0.599	0.648	0.659	0.703	0.657	0.502	0.616
**TEMP**	2 + 2 + 2/2 + 2 + 3	2 + 2 + 4/2 + 3 + 3	2 + 2 + 3/2 + 2 + 3	2 + 2 + 3/2 + 2 + 3	2 + 2 + 3/2 + 1 + 3	2 + 2 + 3/2 + 2 + 3	2 + 2 + 4/2 + 2 + 4
**ATEMP-Eye**	2/2	2/2	2/2	2/2	2/2	2/2	2/1
**DSR**	23-23-23	23-23-23	23-23-23	23-23-23	23-23-23	23-23-23	23-23-23
**KOD**	S + S + K	S + S + K	S + S + K	S + S + K	S + S + K	/	S + K + K
**VS**	158	172	174	173	169	172	166
**SC**	60	51	51	53	49	52	46+
**CP**	1	1	1	1	1	1	1

**Table 5:** Comparisons of main morphological characteristics of *Achalinus ningshanensis occidentalis* ssp. nov. and *A. n. ningshanensis*. S + S + K = smooth on anterior and mid, keeled on posterior body; S + K + K = smooth on anterior, keeled on mid, and posterior body; K + K + K = keeled on anterior, mid and posterior body.

Species	*A. n. occidentalis* ssp. nov.		*A. n. ningshanensis*	
**Sex**	♂	♀	♂	♀
**N**	4	10	2	12
**TL**	178–421	309–493+	363–412	179–577
**TAL**	36–95	48–86+	72–87	28–89
**TAL/TL**	0.202–0.226	0.155–0.178	0.198–0.211	0.121–0.161
**HW/HL**	0.47–0.54	0.44–0.57	0.48–0.52	0.37–0.57
**ED**	0.70–1.56	0.81–1.47	0.7–1.0	0.82–1.33
**MT**	21	21–22	20	20–22
**SL**	6	6	6	6
**IL**	5 or 6	5 or 6	5	4 or 5
**Chin**	2	2	3–4	3–4
**Lor**	1	1	1	1
**LorH/LorL**	0.760–0.946	0.612–1.040	0.544–0.617	0.458–0.681
**LSBI/LSBP**	<	<	=	=
**TEMP**	2 + 2 + 2/3/4	2 + 1/2 + 3/4	2 + 2/3 + 3/4	1/2 + 1/2/3 + 3/4
**ATEMP-Eye**	2	1 or 2	2	1 or 2
**DSR**	23-23-23	23(21)-23(22)-23(21)	23-23-23	23-23-23(21)
**KOD**	S + K + K or S + S + K	S + K + K or S + S + K	K + K + K	K + K + K
**VS**	155–160	165–174	161–165	159–177
**SC**	60–65	49–53	51–56	41–47
**CP**	1	1	1	1

**Table 6:** Morphological characteristics of Achalinus obtained from specimens examined in this study and the literature. Int. fus.: internasal fused to prefrontal; Pre. fus.: prefrontal fused to loreal; TAL/TL-M or F: TAL/TL in males or females; VEN-M or F: VEN in males or females; SC-M or F: SC in males or females; ?: further verification required.

Species	TAL/TL-M	TAL/TL-F	MT	Int fus.	Pre fus.	LorH/LorL	LSBI/LSBP	DSR	SL
*A. n. occidentalis* ssp. nov.	0.202–0.226	0.155–0.178	21–22	0	0	0.61–1.04	<1	23(21)-23(22)-23(21)	6
*A. ater*	0.190–0.220		–	0	0	0.40	>1	(21–23)-(21–25)-(21–25)	6
*A. dabieshanensis*	0.177–0.223	0.168	–	0	0	0.73–0.83	<1	23-23-23	6
*A. damingensis*	0.246	?	–	0	0	0.65	>1	23-23-23	6
*A. dehuaensis*	0.263–0.286	0.206–0.217	31–33	0	0	–	>1	23-23-23	6
*A. emilyae*	?	0.183–0.203	27–28	0	0	–	>1	23-23-23	6
*A. f. chigirai*	0.32		14	0	1	–	=1	(25–27)-(25–27)-25	6
*A. f. formosanus*	0.16		17	0	1(usually)	–	=1	29-27-25	6
*A. hainanus*	?	0.258–0.266	–	0	0	–	=1	23-23-23	6
*A. huangjietangi*	0.197–0.232	0.152–0.158	–	0	0	0.70–0.74	<1	23-23-23	6
*A. hunanensis*	0.221–0.225	?	23	0	0	0.62–0.70	>1	23-23-23	6
*A. jinggangensis*	0.217	0.174	–	0	1	–	>1	23-23-23	6
*A. juliani*	0.264–0.365	0.224	28	0	0	–	>1	25-23-23	6 (7)
*A. meiguensis*	0.142–0.238		17	1	0	–	–	(21–23)-(19–21)-(19–21)	6
*A. nanshanensis*	0.215–0.246	?	18	0	0	0.47–0.53	>	(23–25)-(23–25)-(23–25)	6
*A. niger*	0.151–0.179		–	0	0	0.67	</=1	25-25-23	6
*A. n. ningshanensis*	0.198–0.211	0.121–0.161	20–22	0	0	0.458–0.681	=1	23-23-23(21)	6
*A. panzhihuaensis*	0.246	?	28	1	0	0.67	–	23-23-19	6
*A. pingbianensis*	0.243	0.172	20	0	1	–	=1	23-23-23	7
*A. quangi*	0.283–0.304	0.218–0.262	27–29	0	0	–	>1	(23–25)-23-(21–23)	6
*A. rufescens*	0.191–0.276		23	0	0	0.80–1.00	>1	23-(23–25)-23	6
*A. sheni*	0.183–0.224	0.149–0.164	24–25	0	0	0.53–0.93	=1	23-23-23	6
*A. spinalis*	0.213	0.158–0.275	16–20	0	0	–	</=1	23-(21–23)-(21–23)	6
*A. timi*	0.213	?	27	0	1	–	>1	25-25-23	6
*A. tranganensis*	?	0.254 (+)	29	0	0	–	>1	25-23-23	6
*A. werneri*	0.250–0.300		–	0	0	–	=1	?-(21–23)-?	6
*A. yangdatongi*	0.261–0.262	0.180–0.200	24–26	0	0	0.57	>1	23-23-23	6
*A. yunkaiensis*	0.185–0.200	0.156–0.204	20–24	0	0	0.49–0.64	=1	23-23-23	6
*A. vanhoensis*	0.264	?	32	0	1	–	>1	25-23-23	6/7
*A. zugorum*	0.229	?	28	0	1	–	>1	25-23-23	6
**Species**	**SL-Eye**	**IL**	**Chin**	**TEMP**	**aTEMP-Eye**	**VEN-M**	**VEN-F**	**SC-M**	**SC-F**
*A. n. occidentalis* ssp. nov.	4–5	5 or 6	2	2 + 1/2/3 + 3/4	1–2	155–160	165–174	60–65	49–53
*A. ater*	4–5	5–6	2 or 3	2 + 2 + 3	2	156–170		47–70	
*A. dabieshanensis*	4–5	5	2	2 + 2 + 3/4	2	141–151	155	46–55	45
*A. damingensis*	4–5	6	2	2 + 2 + 3	2	162	?	74	?
*A. dehuaensis*	4–5	5	2	2 + 2/3 + 3/4	1–2	142–147	152–154	74–81	63–65
*A. emilyae*	4–5	5	2	2 + 2 + 3	1	?	157–161	?	56–63
*A. f. chigirai*	4–5	5–6	2 or 3	2 + 2	2	161–167	?	96–97	?
*A. f. formosanus*	4–5	6–7	2 or 3	2 + 2	1	158–176	164–184	62–83	61–70
*A. hainanus*	4–5	5	2	1 + 2 + 3/4	1	?	165–168	?	67–69
*A. huangjietangi*	4–5	5–6	2	2 + 2 + 3/4	2	157–160	170	59–67	47
*A. hunanensis*	4–5	5–6	2	2 + 2 + 4	2	163–165	?	69–72	?
*A. jinggangensis*	4–5	6	2	1/2 + 2 + 3/4	2	156	164	64	51
*A. juliani*	4–5 (5–6)	6	2	2 + 2 + 4	2	163–169	179	91	77
*A. meiguensis*	4–5	6	3	2/3 + 2/3	1	146–173		39–62	
*A. nanshanensis*	4–5	6	2	2 + 2/3 + 4	2	147–158	?	64–71	?
*A. niger*	4–5	6	2 or 3	2 + 2/3	2	169–170	172–185	68–72	52–58
*A. n. ningshanensis*	4–5	5	3 or 4	1/2 + 1/2/3 + 2/3/4	1–2	161–165	159–177	51–56	41–47
*A. panzhihuaensis*	4–5	6	3	2 + 2 + 3	1	160	?	73	?
*A. pingbianensis*	5–6	6	2	2 + 2 + 3	1	164	167	56	48
*A. quangi*	4–5	5	2	2 + 2 + 4	1–2	139–141	141–154	75–84	69
*A. rufescens*	4–5	5	2	1/2 + 2 + 3/4	1–2	132–156	150–158	58–82	56–61
*A. sheni*	4–5	5–6	2	2 + 1/2 + 3/4	2	161–170	172–174	55–61	46–49
*A. spinalis*	4–5	5–6	3	2 + 2/3	1–2	146–168		45–62	
*A. timi*	4–5	6	2	2 + 2 + 3	1	170	?	72	?
*A. tranganensis*	4–5	6	2	2 + 2 + 3	2	?	171	?	73(+)
*A. werneri*	4–5	6	2	2 + 3/4	–	157–171	174–191	90–98	67–85
*A. yangdatongi*	4–5	5–6	2	2 + 2/3 + 2/3	2	155	170–171	76	59–64
*A. yunkaiensis*	4–5	6	2	2 + 2 + 3/4	2	151–162	144–156	49–56	51–55
*A. vanhoensis*	4–5/5–6	6	2	2 + 2 + 3	2	176	?	84	?
*A. zugorum*	4–5	7	2	2 + 2 + 3	2	173	?	70	?

## Data Availability

The data presented in this study are available on request from the corresponding author. ZooBank Code: urn:lsid:zoobank.org:act:75A7E98C-EAB7-4C42-B02D-D0BAE362E3D9; urn:lsid:zoobank.org:pub:DAC31888-5B7F-4C8D-A875-59BD204A25EC.
